# Challenging old microbiological treasures for natural compound biosynthesis capacity

**DOI:** 10.3389/fbioe.2024.1255151

**Published:** 2024-02-01

**Authors:** Imen Nouioui, Alina Zimmermann, Oliver Hennrich, Shuning Xia, Oona Rössler, Roman Makitrynskyy, Juan Pablo Gomez-Escribano, Gabriele Pötter, Marlen Jando, Meike Döppner, Jacqueline Wolf, Meina Neumann-Schaal, Chambers Hughes, Yvonne Mast

**Affiliations:** ^1^ Department Bioresources for Bioeconomy and Health Research, Leibniz Institute DSMZ -German Collection of Microorganisms and Cell Cultures, Braunschweig, Germany; ^2^ German Center for Infection Research (DZIF), Partner Site Tübingen, Tübingen, Germany; ^3^ Department of Microbiology, Biotechnology, Faculty of Science, Interfaculty Institute of Microbiology and Infection Medicine, University of Tübingen, Tübingen, Germany; ^4^ Braunschweig Integrated Centre of Systems Biology (BRICS), Braunschweig, Germany; ^5^ Technische Universität Braunschweig, Institut für Mikrobiologie, Braunschweig, Germany

**Keywords:** actinomycetes, *Streptomyces*, novel species, polyphasic taxonomy, biosynthetic gene cluster, antibiotic

## Abstract

Strain collections are a treasure chest of numerous valuable and taxonomically validated bioresources. The Leibniz Institute DSMZ is one of the largest and most diverse microbial strain collections worldwide, with a long tradition of actinomycetes research. Actinomycetes, especially the genus *Streptomyces*, are renowned as prolific producers of antibiotics and many other bioactive natural products. In light of this, five *Streptomyces* strains, DSM 40971^T^, DSM 40484^T^, DSM 40713^T^, DSM 40976^T^, and DSM 40907^T^, which had been deposited a long time ago without comprehensive characterization, were the subject of polyphasic taxonomic studies and genome mining for natural compounds based on *in vitro* and *in silico* analyses. Phenotypic, genetic, and phylogenomic studies distinguished the strains from their closely related neighbors. The digital DNA–DNA hybridization and average nucleotide identity values between the five strains and their close, validly named species were below the threshold of 70% and 95%–96%, respectively, determined for prokaryotic species demarcation. Therefore, the five strains merit being considered as novel *Streptomyces* species, for which the names *Streptomyces kutzneri* sp. nov., *Streptomyces stackebrandtii* sp. nov.*, Streptomyces zähneri* sp. nov.*, Streptomyces winkii* sp. nov., and *Streptomyces kroppenstedtii* sp. nov. are proposed. Bioinformatics analysis of the genome sequences of the five strains revealed their genetic potential for the production of secondary metabolites, which helped identify the natural compounds cinerubin B from strain DSM 40484^T^ and the phosphonate antibiotic phosphonoalamide from strain DSM 40907^T^ and highlighted strain DSM 40976^T^ as a candidate for regulator-guided gene cluster activation due to the abundance of numerous “*Streptomyces* antibiotic regulatory protein” (SARP) genes.

## 1 Introduction

Microorganisms have long been recognized as a prolific source for valuable bioactive substances (Newman and Cragg, 2016). Especially, actinomycetes are well known for their remarkable biosynthetic potential, with the ability to produce a wide range of natural products, including antibiotics, immunosuppressants, anticancer agents, antifungal compounds, and many other bioactive molecules. Within the family *Streptomycetaceae*, *Streptomyces* is the most prominent genus with respect to the production of bioactive secondary metabolites, including many antibiotics. With over 700 validly named species, *Streptomyces* accounts for more than 50% of all clinically useful antibiotics, including tetracyclines, erythromycin, streptomycin, and vancomycin (Heul et al., 2018). Previous screening campaigns of soil-derived streptomycetes yielded many currently recognized drugs, such as the antibacterial substance streptomycin, the antifungal metabolite nystatin, and the anticancer compound doxorubicin [Schatz et al., 1944; Hazen and Brown, 1951; Acarmone et al., 1969; for review articles, see the work of Genilloud (2017), Baltz (2005), and Berdy (2005)]. However, in the last decades, the discovery rates of novel compounds have declined immensely, which is largely due to the high rediscovery rate of already known substances (Baltz, 2019; van Bergeijk et al., 2020). Classical screening attempts usually employ the so-called Waksman platform, where soil-derived microorganisms are screened for their antimicrobial activity against a panel of bacterial test strains. Re-identification of known substances is partly based on the experimental setup for compound isolation and detection. Standard procedures mostly concentrate on strains producing bioactive compounds readily and in high yields and focus on compounds that show characteristic mass spectrometry patterns (MS) and ultra-violet (UV) spectroscopy signals. Such compounds are commonly referred to as the ‘low-hanging fruits’ of antibiotics research (Panter et al., 2021). However, re-identification of known substances is also a matter of the phylogenetic uniqueness of the producer strain. Phylogenetically related strains tend to produce similar secondary metabolites (Handayani et al., 2021), and correspondingly, it has been shown that phylogenetic uniqueness is correlated with the diversity of novel natural compounds (Hoffmann et al., 2018). Consequently, re-identification of known substances is also a matter of dereplication of known producer strains. Thus, regarding novel compound discovery, it is expedient to focus on phylogenetically novel producer strains.

Even though actinomycetes have been extensively exploited for drug discovery over the past decades, they have continued to be valuable sources for novel antibiotics. Recent advances in genome sequencing technology and large-scale bioinformatics analyses have revealed an enormous genetic potential for the production of yet undiscovered natural compounds. It is expected that only 3% of the overall genomic potential for natural product biosynthesis has been discovered so far and that the genus *Streptomyces*, especially, represents a huge untapped reservoir for the production of novel secondary metabolites (Gavriilidou et al., 2022). The capability to produce antibiotics is genetically encoded in the actinomycetes’ genomes, whereby the corresponding genes are organized as biosynthetic gene clusters (BGCs) (Medema et al., 2015). *Streptomyces* genomes harbor, on average, around 40 BGCs (Belknap et al., 2020), with the majority of all clusters (∼90%) being proposed as cryptic or silent, which means that the encoded antibiotics are not known or the respective substances are not produced under standard lab conditions, respectively (Walsh and Fischbach, 2010). State-of-the-art discovery efforts make use of this genetic potential and apply genome mining and genetic engineering techniques to access the genetically encoded biosynthetic potential of microbial producer strains. In recent years, this has already led to the successful discovery of novel potent antibiotics, as recently demonstrated by the identification of the macolacins and cilagicins, which have been deduced from biosynthetic genes primarily (Wang et al., 2022a; b).

In the effort to find novel antibiotics, microbial culture collections represent a treasure trove of pure, well-curated, and freely accessible strains that can be used for bioprospecting. In general, microbial culture collections are dedicated to collecting, maintaining, and distributing microbial strains among microbiologists, as well as being committed to preserving microbial diversity. Thereby, strain collections are essential resources for microbiology, biotechnology, and many other fields and provide a valuable source of microorganisms for research, education, bioprospecting, and conservation. Researching large strain collections has the advantage of circumventing laborious strain isolation efforts and also allows access to many strains whose genome sequences are already available. The Leibniz Institute DSMZ-German Collection of Microorganisms and Cell Cultures is one of the largest and most diverse strain collections, housing bioresources from over 90 countries all over the world and 80% of all reported microbial type strains (https://www.dsmz.de/dsmz; https://www.sciencetheearth.com/latest-blog/the-deutsche-sammlung-von-mikrooganismen-und-zellkulturen-gmbh-dsmz-in-braunschweig-germany). The DSMZ subcollection “Actinobacteria” harbors more than 4,000 actinomycetes containing >2,000 type strains and including ∼2,500 streptomycetes, with genome sequence data available for ∼800 actinobacteria. Many strains included in culture collections have been deposited a long time ago without comprehensive characterization.

In this study, we report on the identification and description of five novel species from the DSMZ strain collection belonging to the genus *Streptomyces*. We analyzed their genetic potential for the biosynthesis of bioactive natural compounds and show empirical evidence for the production of known and novel bioactive natural products.

## 2 Materials and methods

### 2.1 Bacterial strains and cultivation conditions

Strains DSM 40907^T^, DSM 40484^T^, DSM 40976^T^ (=Gütt 467), and DSM 40971^T^ (= Sandoz 59283) were isolated from soil samples of unknown countries and deposited in the German Collection of Microorganisms and Cell Cultures (DSMZ) before the 1980s. Strain DSM 40713^T^ (=Tü 43 = ETH 21510) was isolated from the soil sample collected in Switzerland and deposited by Professor Dr. Hans Zähner (Eberhard Karls University of Tübingen, Germany) in the DSMZ open culture collection. Active culture of the strains and their close phylogenetic neighbors, including *Streptomyces nojiriensis* DSM 41655T, *S*. *gardneri* DSM 40064T, *S. gardneri* DSM 40016T, *S. cacaoi asoensis* DSM 41440T, *S. marinus* DSM 41968T, and *S*. *tauricus* DSM 40560T, were maintained on medium DSMZ 65 (GYM, glucose, yeast, and malt extracts). The purity of the cultures was checked using light microscopy. For chemotaxonomic characterization, the cultures were prepared on ISP 2 broth [International *Streptomyces* Project (ISP); Shirling and Gottlieb, 1966] medium shaken at 200 rpm for 7 days at 28°C. The biomasses were harvested by centrifugation at 4,000 rpm for 15 min, washed with sterile distilled water, and freeze-dried. Fatty acid analysis was carried out from wet biomass collected from a 7-day-old culture prepared on ISP 2 broth medium.

### 2.2 Cultural and growth properties

The cultural properties of the strains were recorded on ISP 1 (DSMZ 1764), ISP 2 (DSMZ 987), ISP 3 (DSMZ 609), ISP 4 (DSMZ 547), ISP 5 (DSMZ 993), ISP 6 (DSM 1269), ISP 7 (DSM 1619), nutrient (DSMZ 1), Bennett’s (DSMZ 548), and trypticase soy (DSMZ 535) agar media after 7 days of incubation at 28°C. The growth of the strains was tested under a wide range of temperatures (4°C, 10°C, 15°C, 20°C, 25°C, 28°C, 37°C, 42°C, and 45°C) and at various pH values (5.0, 5.5, 6.0, 7.5, 8.0, 8.5, 10.0, and 12.0) in DSMZ 65 medium. All these tests were carried out in duplicate using a bacterial suspension of 5 on the McFarland scale. The color of the aerial and substrate mycelium as well as the diffusible pigments were compared against color charts.

### 2.3 Phenotypic and chemotaxonomic properties

Chemotaxonomic markers of the strains and their close phylogenomic neighbors were determined using standard thin-layer chromatographic procedures. To this end, isomers of diaminopimelic acid (A2pm) (Schleifer and Kandler, 1972) and polar lipid (Bligh and Dyer, 1959; Tindall et al., 2007) patterns were carried out. Cellular fatty acids of the strains were extracted and analyzed by gas chromatography (Agilent 6890N) following the standard protocols of the microbial identification (MIDI) system (Sasser, 1990). Fatty acids were identified by a GC–MS run on an Agilent GC–MS 7000D instrument (Vieira et al., 2021). Isoprenoid quinones were extracted, separated by HPLC, and identified by using both a DAD and high-resolution mass spectrometer, according to the work of Schumann et al. (2021). Biochemical and enzymatic properties of the strains and their phylogenomic neighbors were determined using API-ZYM and API 20NE strips, as instructed by the manufacturer (bioMerieux, Lyon, France).

### 2.4 Molecular identification and genome sequencing

Wet biomass, harvested from a 7-day-old culture on ISP 2 broth medium, was used for genomic DNA preparation for single gene and genomic analyses, as reported previously (Zimmermann et al., 2023). PCR-mediated amplification of the 16S rRNA gene was performed with universal primers 7F (5′-AGA GTT TGATC(AC)TGG CTC AG-3′) and 1492R (5′-ACGG(CT)TAC CTT GTT ACG ACTT-3′), according to the work of Weisburg et al. (1991). The PCR products (∼1520 bp) were sequenced using the fluorescence-based Sanger method (Sanger et al., 1977) with primers 7F and 1492R. The resulting 16S rRNA gene sequences were used for phylogenetic studies (below). The five strains described in this work were selected on the basis of 16S rRNA-based phylogenetic uniqueness for whole shotgun genome sequencing. Illumina genome sequencing and assembly, based on 250-bp paired-end reads from an ∼500-bp insert library, was outsourced to MicrobesNG (Birmingham, United Kingdom). Draft genome sequences were annotated with the RAST-SEED webserver (https://rast.nmpdr.org/) (Overbeek et al., 2014).

### 2.5 Phylogeny and comparative genomic studies

The almost complete 16S rRNA gene sequence (>1,400 bp) of the strains and their close phylogenetic relatives were used in the present study. Pairwise 16S rRNA gene sequence similarities between the strains and their close phylogenetic neighbors were estimated under the setting recommended by Meier-Kolthoff et al. (2013a), which is implemented in the phylogeny web server available in Genome-to-Genome Distance Calculator (GGDC) 2.1 (http://ggdc.dsmz.de) (Meier-Kolthoff et al., 2022). The reference strains were retrieved from the EzBioCloud server (https://www.ezbiocloud.net/) (Yoon et a., 2017).

The maximum-likelihood (ML) trees based on 16S rRNA gene and genome sequences of the strains were carried out with the Type (Strain) Genome Server (TYGS) v. 1.0 (https://tygs.dsmz.de/), a free bioinformatics tool for whole-genome-based taxonomic analysis (Meier-Kolthoff and Göker, 2019). Phylogenetic classification using the TYGS is based on a genome database that contains the genomic, taxonomic, and nomenclatural data of all currently available type strains. The database is constantly updated. The TYGS platform allows phylogenetic analyses based on the full-length genome sequence of the strain of interest, which is compared with a database of type strain genomes. Thereby, the TYGS provides information on the similarity of the strain to its nearest related type strain with the help of digital DNA–DNA hybridization (dDDH) values calculated by the GGDC 2.1 (http://ggdc.dsmz.de) (Meier-Kolthoff et al., 2013b) and determines differences in genomic G + C contents. dDDH and average nucleotide identity (ortho ANI) between the strains and their phylogenomic relatives were determined using the GGDC webservers, with the recommended formula 4 and the ANI calculator from EzBioCloud (https://www.ezbiocloud.nettools/ani), respectively.

### 2.6 *In silico* screening for secondary metabolites

The antiSMASH web tool v. 6.0 (Blin et al., 2021) was used to analyze whole-genome sequences of the strains and their closest phylogenomic neighbors for the presence of BGCs. Gene cluster similarity is displayed in percentage and indicates the number of genes similar to a known cluster. Genes are similar if a BLAST alignment yields an *e-*value < 1 × 10^−5^ and if sequence identity is >30%. In addition, the shortest alignment must encompass >25% of the sequence. If all genes of a known cluster can be found in the query cluster, the sequence similarity is 100%. The similarity decreases if fewer genes of the known cluster can be found in the query cluster (Medema et al., 2011). The default settings were used for all analyses.

### 2.7 Fermentation and preparation of culture extracts

The strains DSM 40484^T^, DSM 40713^T^, DSM 40907^T^, DSM 40971^T^, and DSM 40976^T^ were cultivated by inoculation from a GYM plate in 50 mL medium R5 (103 g L^−1^ sucrose, 10 g L^−1^ glucose, 0.25 g L^−1^ K_2_SO_4_, 10.12 g L^−1^ MgCl_2_ x 6 H_2_O, 0.1 g L^−1^ casamino acids, 5 g L^−1^ yeast extract, 5.73 g L^−1^ TES (pH 7.2), 2.94 g L^−1^ CaCl_2_ x 2H_2_O, 0.05 g L^−1^ KH_2_PO_4_, 3 g L^−1^ L-proline, and 2 mL trace element solution, according to the work of Handel et al. (2022); pH 7.4) at 28°C in 250 mL Erlenmeyer flasks on an orbital shaker (180 rpm). After 2 days of cultivation, 5 mL of the preculture was used to inoculate 250 mL Erlenmeyer flasks with 50 mL of production media R5 or NL 800 (5 g L^−1^ glucose, 10 g L^−1^ glycerol, 10 g L^−1^ soluble starch, 58 g L^−1^ oatmeal, 2 g L^−1^ yeast extract, 1 g L^−1^ NaCl, and 1 g L^−1^ CaCO_3_; pH 7.2) as production cultures, which were incubated for 3 days at 28°C on an orbital shaker at 180 rpm. For extraction of organic compounds, 5 mL of culture was harvested after 3 days and extracted with 5 mL ethyl acetate (EtAc) for 3–6 h at room temperature under constant vertical rotation. After centrifugation at 5,000 rpm for 10 min, the organic phase was completely dried using a centrifugal evaporator (SP Genevac EZ-2, “Low BP” program). The concentrated extracts were dissolved in 0.25 mL 50% methanol (MeOH), resulting in a 20-fold concentrated EtAc extract sample. The crude extracts were used for bioassays and liquid chromatography–high-resolution mass spectrometry (LC–HRMS) analysis.

### 2.8 Bioassays

The crude extracts were analyzed for antimicrobial activities using agar-well diffusion assays against a panel of Gram-positive and Gram-negative bacteria, yeast, and fungal reference strains: *Staphylococcus aureus* DSM 18827, *Enterococcus faecium* DSM 20477^**T**^, *Pseudomonas aeruginosa* DSM 1117, *Escherichia coli* DSM 1103, *Proteus vulgaris* DSM 2140, *Candida albicans* DSM 1386, and *Trichophyton rubrum* DSM 16111. Active culture of these strains was prepared under the growth conditions recommended by the DSMZ collection (https://www.dsmz.de/collection/catalogue). Bioassay test plates were prepared as reported previously (Handayani et al., 2021). A volume of 30 µL methanolic crude extract from three independent biological samples of the five *Streptomyces* strains DSM 40484^T^, DSM 40713^T^, DSM 40907^T^, DSM 40971^T^, and DSM 40976^T^ was pipetted in prepared agar wells of bioassay test agar plates inoculated with the test strains listed above. The plates were incubated overnight at 28°C and 37°C according to the optimal growth temperature of the bioassay test strains (*T. rubrum* bioassays plates were incubated at room temperature in darkness for several days, with daily observation). Antibiotic activity was estimated by measuring the diameter of the inhibition zone.

### 2.9 Chemical analyses for compound detection

Extracts were analyzed on an analytical 1290 Infinity II LC system coupled to a Bruker Impact II QTOF mass spectrometer. Gradient elution was performed through a C18 porous core-shell column (Phenomenex Kinetex C18, 100 × 2.1 mm, 1.7 μm, 100 Å) using a 10-min gradient from 5% MeCN to 100% MeCN in water, supplemented with 0.1% formic acid. The flow rate was 0.5 mL min^−1^. QTOF parameters were as follows: 150–2,000 m*/z* scan range, 4,500 V capillary voltage, 500 V end plate offset, 2.8 bar nebulizer, 220°C drying temperature, and 10 L min^−1^ nitrogen drying gas.

Identification of cinerubin: Cinerubin B (t_R_ = 5.7 min) was initially identified using a combination of its UV/Vis spectrum and LC–HRMS. The UV/Vis spectrum (λ_max_ = 235, 255, 290, and 490 nm) matched cinerubin B in an “in-house” UV/Vis database. Its protonated molecular ion *m/z* 826.3278 [M + H]^+^ (calculated (calcd) for C_42_H_52_NO_16_, 826.3281, Δ 0.3 ppm) yielded a formula (C_42_H_51_NO_16_) matching that of cinerubin B.

Identification of phosphonoalamide: Phosphonoalamides were identified using HRMS. A (t_R_ = 0.5 min): *m/z* 340.1270 [M + H]^+^ (calcd for C_11_H_23_N_3_O_7_P, 340.1268, Δ −0.5 ppm); B (t_R_ = 0.5 min): *m/z* 370.1378 [M + H]^+^ (calcd for C_12_H_25_N_3_O_8_P, 370.1374, Δ −1.0 ppm); C (t_R_ = 0.8 min): *m/z* 354.1424 [M + H]^+^ (calcd for C_12_H_25_N_3_O_7_P, 354.1425, Δ 0.2 ppm); and D (t_R_ = 0.9 min): *m/z* 368.1582 [M + H]^+^ (calcd for C_13_H_27_N_3_O_7_P, 368.1581, Δ −0.3 ppm). The MS/MS fragmentation data for the detected compounds matched the data reported by Kayrouz et al. (2020).

### 2.10 Inactivation of a cinerubin B biosynthesis gene in DSM 40484^T^

To inactivate *ctg27_10* from the predicted cinerubin BGC, a 0.64-kb-long DNA fragment, harboring an internal part of the gene, was amplified from genomic DNA by PCR with primers RM_ctg27_10_kn_vn_for (AAA​AAA​GCT​TTG​AAA​CTG​GAG​GAG​GAG​TAC) and RM_ctg27_10_kn_vn_rev (AAA​AAA​GAA​TTC​GTC​TCG​TGG​ATG​TCG​TTC​TG). The PCR product was digested with *Hin*dIII and *Eco*RI and then cloned into the respective restriction sites of pKC1132, a suicide vector containing an apramycin resistance gene, to generate pKC1132/ctg27_10_kn. The resulting plasmid was transferred to DSM 40484^T^ by conjugation using *E. coli* ET12567/pUZ8002 as a donor strain. Exconjugants were selected by resistance to apramycin (indicative of single crossover), leading to the creation of the mutant strain DSM 40484^T^ *ctg27_10::pKC1132/ctg27_10_kn* (*Mctg27_10*).

### 2.11 SARP overexpression in the strain DSM 40976^T^

A detailed analysis of all BGCs from the five strains revealed the presence of numerous SARP genes in different BGCs of the strain DSM 40976^T^. The latter was the subject of SARP overexpression studies. In this context, a conjugative, self-replicative *papR2* expression construct was generated. The plasmid pGM1190 was used as a vector, which contained the *tipA* promoter for the induction of gene transcription, and *oriT*, which is required for the intergenic conjugation from *E. coli* to *Streptomyces*. pGM1190 is a multi-copy and very stable plasmid, which does not require antibiotic selection in production cultures (Muth, 2018). The *papR2* gene was excised as an ∼1 kb *Hin*dIII/*Nde*I-fragment from pRM4/*papR2* (Krause et al., 2020) and cloned into the *Hin*dIII/*Nde*I-linearized ∼6.9 kb pGM1190 vector. This resulted in the plasmid pGM1190/*papR2-tipA*p, where *papR2* transcription is under the control of the thiostrepton-inducible *tipA* promoter. The pGM1190/*papR2-tipA*p plasmid was transferred to *Streptomyces* sp. DSM 40976^T^ by conjugation, as described by Kieser et al. (2000), resulting in the strain DSM 40976^T^ pGM1190/*papR2-tipA*p. Apramycin (50 μg/mL) was used for selection when appropriate. To test for the effect of *papR2* expression on secondary metabolite production, the strain DSM 40976^T^ pGM1190/*papR2-tipA*p and the WT strain were each cultivated in OM medium (20 g L^−1^ oat meal, 5 mL trace element solution, pH 7.3; according to Handel et al. (2022)) at 28°C; 20 mL of culture was harvested after 72 h of cultivation. For extraction of organic compounds, cultures were treated as described above. For the preparation of supernatant samples, 20–25 mL from the original 50 mL culture was transferred to a 50 mL Falcon tube and centrifuged at 5,000 rpm for 15 min. The supernatant was concentrated with the centrifugal evaporator (SP Genevac EZ-2, “aqueous” program) to 3–5 mL, resulting in a 5–7-fold concentrated supernatant sample. The crude extract and supernatant samples were analyzed in bioassays against *Micrococcus luteus* (valid name: *Kocuria rhizophila* DSM 11926^T^), as described by Handayani et al. (2021). Experiments were carried out as three independent biological replicates.

## 3 Results and discussion

### 3.1 Polyphasic taxonomic studies

#### 3.1.1 Cultural, morphological, and phenotypic properties

The strains showed phenotypic and morphological features consistent with their classification in the genus *Streptomyces* (Kämpfer, 2012). As shown in Supplementary Table S1, most of the strains grow well on ISP 3, ISP 7, and Bennett’s agar media forming aerial hyphae, which varied from white to gray after 7 days of incubation at 28°C (https://www.dsmz.de/collection/catalogue). All the studied strains grew well on ISP3, ISP 7, DSMZ 65, R5, and Bennett’s media. The strains were able to grow at an incubation temperature between 25°C and 28°C and at a pH of 6–8, and they grew optimally at 28°C and pH 6.5–7 (Supplementary Figure S1; Supplementary Table S1). No growth of the strains was observed at incubation temperature above 37°C and below 10°C. More details about the growth and cultural properties of the strains are provided in Supplementary Table S1. The strains can be distinguished from one another and from their closest phylogenomic relatives by a range of biochemical and enzymatic properties, as shown in Table 1. Strain DSM 40907^T^ was able to produce *α*-glucosidase and lipase C14, unlike its closest relative *S. nijoriensis* DSM 41655^T^. Strain DSM 40713^T^ can be distinguished from the reference strain DSM 41440^T^ by the production of lipase C14, α-chymotrypsin, β-glucosidase**,** and n-acetyl-*β*-glucosaminidase. However, strain DSM 40971^T^ can be differentiated from its close neighbor, *S. marinus*, by its inability to produce α-galactosidase, β-galactosidase, and β-glucuronidase. Qualitative variation in the enzymatic profile of strain DSM 40976^T^ and its close relatives, *S. gardneri* and *S. narbonensis*, was noted, as shown in Table 1. Strain DSM 40484 ^T^ could be distinguished from the type strain DSM 40560^T^ by its ability to produce esterase (lipase) C8, lipase C14, trypsin, and α-chymotrypsin; further biochemical characteristics are displayed in Table 1. Excellence congruence was obtained for all the duplicated phenotypic tests.

**TABLE 1:** Phenotypic properties of the strains and their closest phylogenomic neighbors.

Substrates	DSM 40907^T^	DSM 41655 ^T^	DSM 40976 ^T^	DSM 40064 ^T^	DSM 40016^T^	DSM 40713^T^	DSM 41440^T^	DSM 40484^T^	DSM 40560^T^	DSM 40971^T^	DSM 41968^T^
**API Zym**											
Esterase C4	**+**	**+**	**+**	**+**	**+**	**+**	**+**	**+**	**+**	**+**	**+**
Esterase (lipase) C8	**+**	**+**	**+**	**+**	**+**	**+**	**+**	**+**	**-**	**+**	**-**
Lipase C14	**+**	**-**	**+**	**+**	**-**	**+**	**-**	**+**	**-**	**+**	**+**
Trypsin	**+**	**+**	**+**	**+**	**+**	**+**	**+**	**+**	**-**	**+**	**+**
α-Chymotrypsin	**+**	**+**	**+**	**+**	**-**	**+**	**-**	**+**	**-**	**+**	**+**
Acid phosphatase	**+**	**+**	**+**	**+**	**-**	**+**	**+**	**+**	**+**	**+**	**+**
Naphthol-AS-BI-phosphohydrolase	**+**	**+**	**+**	**+**	**-**	**+**	**+**	**+**	**+**	**+**	**+**
α-Galactosidase	**-**	**-**	**-**	**-**	**-**	**-**	**+**	**-**	**+**	**-**	**+**
β-Galactosidase	**-**	**-**	**+**	**+**	**-**	**+**	**+**	**+**	**+**	**-**	**+**
β-Glucuronidase	**-**	**-**	**-**	**-**	**-**	**-**	**-**	**-**	**-**	**-**	**+**
α-Glucosidase	**+**	**-**	**+**	**+**	**+**	**-**	**-**	**+**	**+**	**+**	**+**
β-Glucosidase	**+**	**+**	**+**	**+**	**+**	**+**	**-**	**+**	**+**	**+**	**+**
n-Acetyl-β-glucosaminidase	**+**	**+**	**+**	**+**	**+**	**+**	**-**	**+**	**+**	**+**	**+**
α-Mannosidase	**-**	**-**	**-**	**+**	**-**	**-**	**-**	**+**	**+**	**+**	**+**
**API 20NE**											
Tryptophan	**+**	**-**	**-**	**-**	**-**	**-**	**-**	**-**	**-**	**-**	**-**
Urea	**-**	**+**	**+**	**+**	**+**	**+**	**-**	**-**	**-**	**-**	**+**
Gelatine	**+**	**+**	**-**	**+**	**+**	**+**	**+**	**-**	**+**	**+**	**+**
*p*-Nitro-phenyl-*β*-D-galactopyranosid	**-**	**-**	**+**	**+**	**+**	**+**	**+**	**+**	**+**	**-**	**+**
Glucose	**+**	**+**	**+**	**+**	**+**	**+**	**+**	**+**	**+**	**-**	**+**
Arabinose	**+**	**+**	**+**	**+**	**-**	**+**	**+**	**+**	**+**	**-**	**+**
Mannose	**+**	**+**	**+**	**-**	**-**	**+**	**+**	**+**	**+**	**-**	**+**
Mannitol	**-**	**+**	**-**	**-**	**-**	**+**	**+**	**+**	**+**	**-**	**+**
N-Acetylglucosamin	**+**	**+**	**+**	**+**	**+**	**+**	**+**	**+**	**+**	**-**	**+**
Maltose	**-**	**+**	**+**	**+**	**+**	**+**	**+**	**-**	**-**	**-**	**+**
Gluconate	**+**	**+**	**+**	**+**	**-**	**+**	**+**	**+**	**+**	**-**	**+**
Adipate	**-**	**+**	**-**	**-**	**-**	**-**	**+**	**-**	**+**	**-**	**+**
Malate	**+**	**+**	**+**	**+**	**-**	**+**	**+**	**+**	**+**	**-**	**+**
Citrate	**-**	**+**	**-**	**+**	**-**	**+**	**+**	**+**	**+**	**-**	**+**
Phenylacetate	**+**	**-**	**+**	**+**	**-**	**-**	**+**	**-**	**-**	**-**	**-**

All the strains were able to produce alkaline phosphatase, leucine arylamidase, valine arylamidase, and cystine arylamidase. All the strains were unable to produce α-fucosidase.

All the strains had a positive reaction to esculin and a negative reaction to arginine, glucose, and caprate.

#### 3.1.2 Chemotaxonomic features

The studied strains and their close phylogenomic neighbors, *S. nojiriensis* DSM 41655^T^, *S. cacaoi* subsp. *asoensis* DSM 41440^T^, *S. gardneri* DSM 40064^T^, *S. narbonensis* DSM 40016^T^, *S. tauricus* DSM 40560^T^, and *S. marinus* DSM 41968^T^, have *LL*-A2pm as the diamino acid of the wall peptidoglycan. The polar lipid profile of the strains and their closest relatives contained diphosphatidylglycerol (DPG), phosphatidylethanolamine (PE), and phosphatidylinositol (PI), as well as unidentified lipids (L), phospholipids (PLs), aminolipids (ALs), glycolipids (GLs), and glycophospholipids (GPLs), as shown in Supplementary Figure S2. Quantitative and qualitative variations in the isoprenoid profiles were detected between the studied strains and their close phylogenetic relatives. The predominant menaquinone (≥15%) of the strains DSM 40907^T^ and DSM 40713^T^ and their closest neighbors, *S. nojiriensis* DSM 41655^T^ and *S. cacaoi* subsp. *asoensis* DSM 41440^T^, was MK-9 H_4_ and MK-9 H_6_. Nevertheless, strains DSM 40976^T^ and its close relatives, *S. gardneri* DSM 40064^T^ and *S. narbonensis* DSM 40016^T^, had MK-9 H_6_ and MK-9 H_8_. Strain DSM 40971^T^ had MK-9 H_4_ and MK-9 H_6_, while strain DSM 41968^T^ contained MK-9 H_6_ and MK-9 H_8_. The quinone pattern of strain DSM 40484^T^ was composed of MK-9 H_4_ and MK-9 H_6_ (Supplementary Table S2). The major fatty acid (>10%) of strain DSM 40907^T^ was C_15:0_ *iso,* C_15:0_ *anteiso,* C_16:0_ *iso,* and C_16:0_, while *S. nojiriensis* DSM 41655^T^, its closest phylogenomic neighbor, had C_15:0_ *iso,* C_15:0_ *anteiso,* and C_16:0._ Strain DSM 40976^T^ had C_17:0_ *anteiso,* C_15:0_ *anteiso,* and C_16:0._ However, the type strains of *S. gardneri* and *S. narbonensis* had C_16:0_ *iso* in addition. The fatty acid profile of strain DSM 40713^T^ consisted of C_15:0_ *iso,* C_15:0_ *anteiso,* C_16:0_ *iso,* C_16:0_, and C_17:0_ *anteiso,* while C_15:0_ *iso,* C_16:0_ *iso,* and C_17:0_ *anteiso* were below the 10% for its close relative, *S. cacaoi* subsp. *asoensis* DSM 41440^T^. Strain DSM 40971^T^ differed from its closest neighbor, *S. marinus* DSM 41968^T^, by a fatty acid pattern composed of C_15:0_ *iso,* C_15:0_ *anteiso,* C_16:0_ *iso,* and C_16:0_, whereas strain DSM 41968^T^ only showed traces of C_16:0_. Strain DSM 40484^T^ had C_15:0_ *iso,* C_15:0_ *anteiso,* C_16:0_ *iso,* C_16:0_, and C_17:0_ *iso*, which is a fatty acid profile similar to that of its relative strain DSM 40560^T^ (Supplementary Table S3).

#### 3.1.3 Phylogeny based on 16S rRNA gene and whole-genome sequences

The 16S rRNA gene sequence similarities between the strains and their closest relatives ranged from 99.3% to 100% (Supplementary Table S4), which were values well above the threshold of 98.65% for prokaryotic species delineation (Kim et al., 2014). Strain DSM 40907^T^ showed a 16S rRNA gene sequence similarity of 100% to that of the type strains of *Streptomyces spororaveus*, *S*. *xanthophaeus*, and *S*. *nojiriensis* species (Supplementary Table S4). Moreover, strain DSM 40976^T^ shared a similar sequence with *S*. *gardneri* NBRC 12865^T^ (99.9%), *S*. *narbonensis* NBRC 1280^T^ (99.9%), and *S*. *zaomyceticus* NBRC 13348^T^ (99.9%). Strains DSM 40484^T^ and DSM 40971^T^ showed a 16S rRNA gene sequence similarity value of 99.3% with *S*. *glomeroaurantiacus* NBRC 15418^T^, *S*. *aurantiacus* NBRC 13017^T^, and *S*. *nanshensis* SCSIO 01066^T^. Strain DSM 40713^T^ had a high 16S rRNA gene sequence similarity to *S*. *humidus* NBRC 12877^T^ (99.7%) and *Streptomyces cacaoi* subsp. *asoensis* NRRL B-16592^T^ (99.5%). Low sequence similarities of 95.0%–98.7% were found among the studied strains.

**FIGURE 1:**
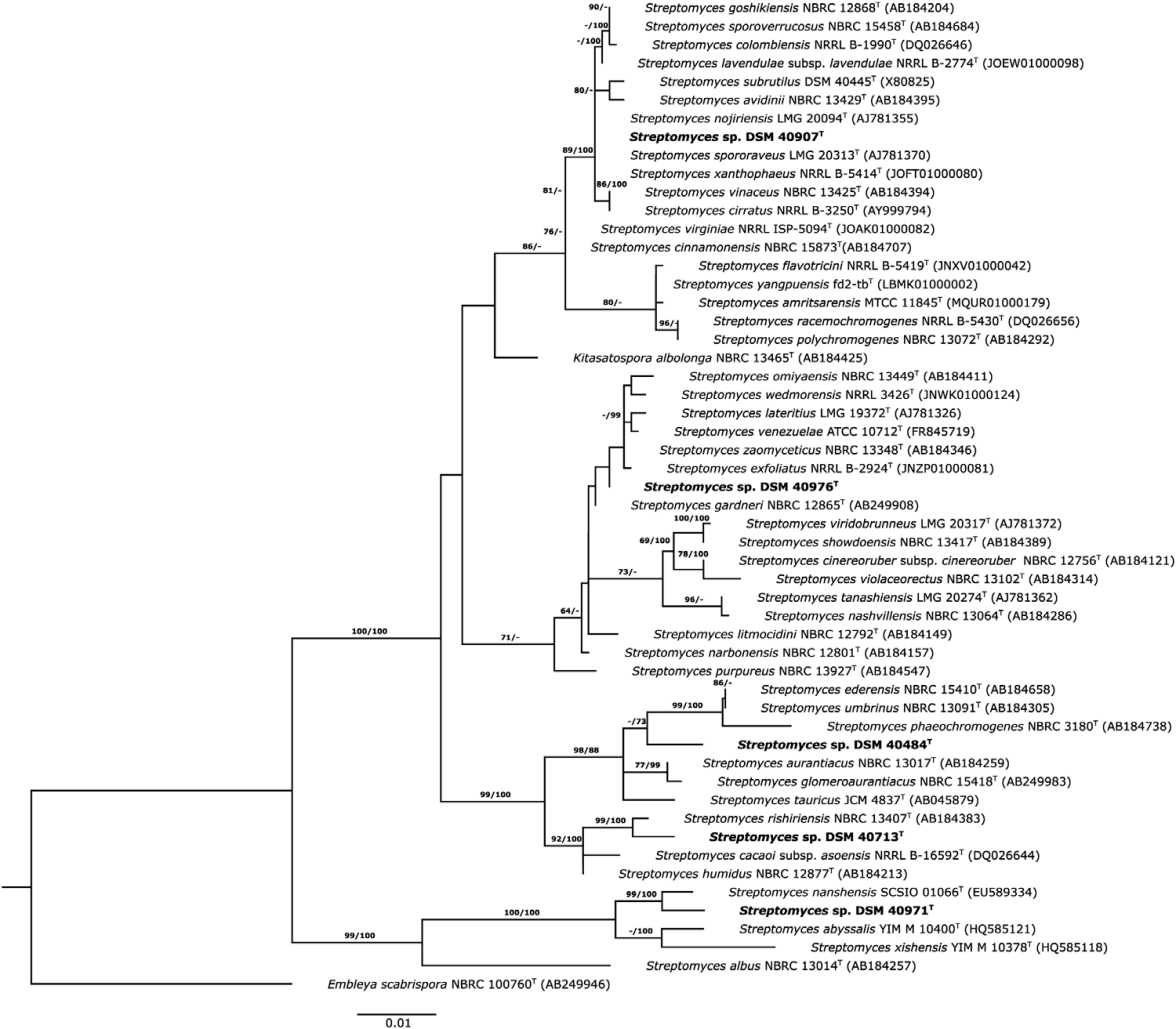
Maximum likelihood phylogenetic tree based on 16S rRNA gene sequences, showing the relationships between the strains and their closely related species. The tree was inferred with FastME from GBDP distances calculated from 16S rDNA gene sequences. The numbers above branches are GBDP pseudo-bootstrap support values > 60% from 100 replications. The tree was rooted at the midpoint.

**FIGURE 2:**
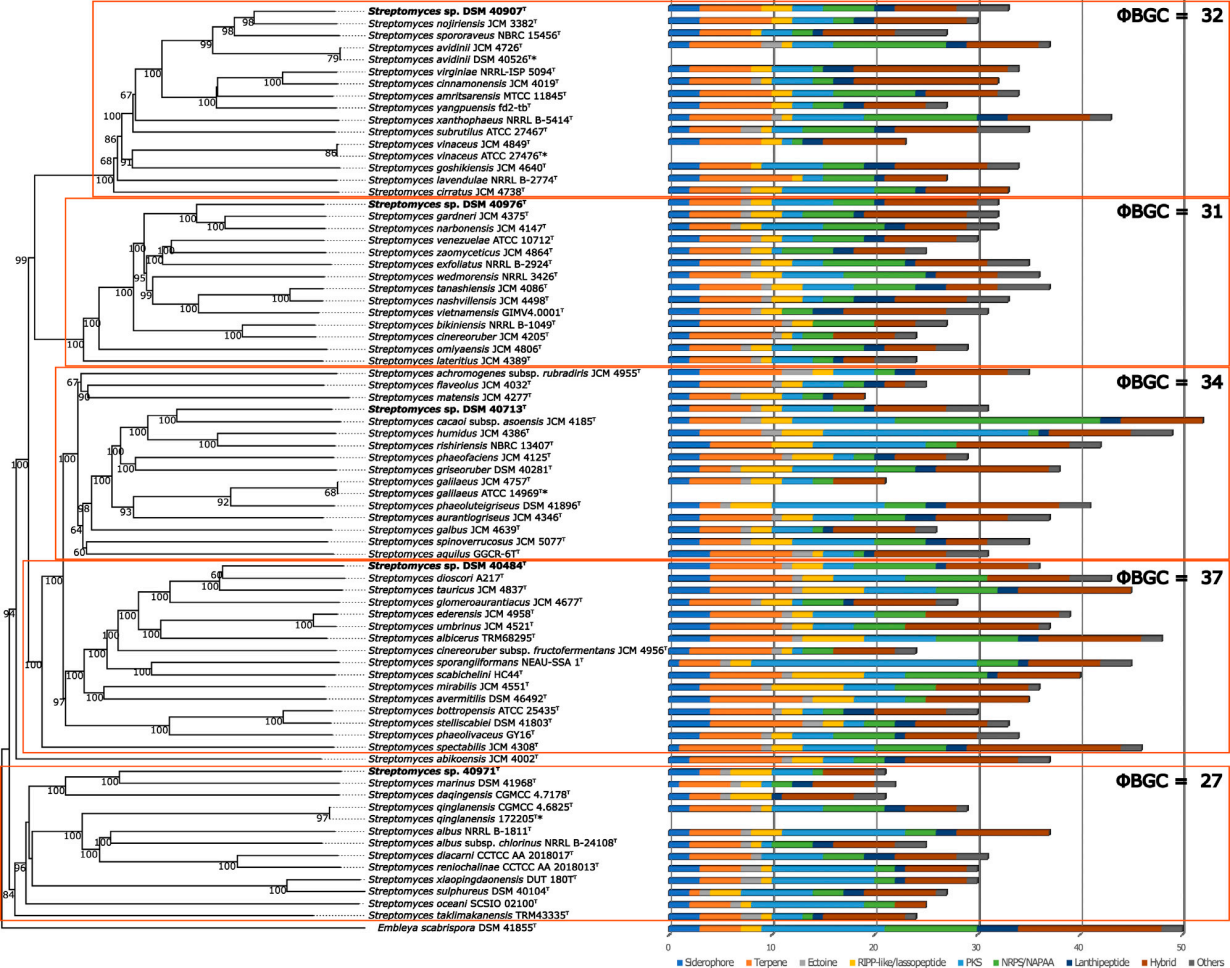
Whole-genome sequence tree generated with the TYGS web server for five novel *Streptomyces* species and closely related species. The tree was inferred with FastME from GBDP distances calculated from genome sequences. The branch lengths are scaled in terms of GBDP distance formula *d*_*4*_. Five distinct clades are highlighted with orange boxes. BGC content is shown at the right side of the tree, and BGC classes are indicated with colors according to the legend shown below. * indicates same strains.

These results were in accordance with the phylogenetic position of the strains in the ML tree, where the strains were distributed in different clusters (Figure 1). Strain DSM 40713^T^ formed a well-supported subcluster with *S. rishiriensis* NBRC 13407^T^ (99.4%) next to the representative strains of *S. humidus* and *S. cacaoi* subsp. *asoensis*. However, strain DSM 40484^T^ occupied a distinct branch loosely associated to a subcluster that encompassed *S. phaeochromogenes* (98.8%), *S. umbrinus* (98.8%), and *S. ederensis* (99.0%) and was next to the type strain of *S. glomeroaurantiacus* and *S. aurantiacus* species. In another well-supported lineage, strain DSM 40971^T^ was grouped with its close relative, *S. nanshensis*. Nevertheless, strain DSM 40976^T^ appeared in a distinct branch loosely associated to the type strain of *S. gardneri* and *S. zaomyceticus* and distant from *S. narbonensis,* which was inserted in another subcluster. Strain DSM 40907^T^ and its close neighbors mentioned above resided in the same branch and cannot be phylogenetically differentiated from one another. It is clear that the resolution of the 16S rRNA gene sequence does not allow reliably distinguishing closely related species (Nouioui et al., 2018; Nouioui and Sangal, 2022). To overcome this limitation, a phylogenomic tree on the whole-genome sequence was constructed, and the taxonomic status of these strains was resolved. Strain DSM 40907^T^ was grouped with *S. nojiriensis* and *S. spororaveus*, with the former as the closest phylogenomic neighbor. Strain DSM 40976^T^ was found to be closely related to a subcluster that contained the type strains of *S. gardneri* and *S. narbonensis* species. Strain DSM 40713^T^ formed a well-supported subcluster with *S. cacaoi* subsp. *asoensis* next to *S. humidus* and *S. rishiriensis.* Strain DSM 40484^T^ was found to be closely related to *‘Streptomyces dioscori* A127’ and *S. tauricus* JCM 4837^T^. However, *‘Streptomyces dioscori*’ has no standing in nomenclature as the name has not been validly published since 2018. Therefore, only *S. tauricus* can be considered the closest phylogenomic neighbor to strain DSM 40484^T^. Strain DSM 40971^T^ was closely related to *S*. *marinus*, together forming a well-supported subgroup associated with *S*. *daqingensis* CGMCC 4.7178^T^ (Figure 2).

#### 3.1.4 Genomic features and comparative genomic analysis

The genomic features of the five studied strains were consistent with those of the genus *Streptomyces* (Kämpfer, 2012). The strains had a genome size between 7.2 and 9.2 Mbp, a G + C content of 70.8%–72.0%, a number of coding sequences from 6,369 to 8,959, and a number of RNAs between 58 and 84 (Supplementary Table S5). The dDDH values between the genome sequence of strains DSM 40907^T^, DSM 40976^T^, DSM 40713^T^, DSM 40484^T^, and DSM 40971^T^ were well below the 70% threshold established for prokaryotic species demarcation (Wayne et al., 1987), indicating the assignment of each of the strains to a new species as they could be distinguished from their close phylogenomic relatives on this basis (Table 2). The results were coherent with the ANI scores, which were below the cut-off point of 95%–96% for species delineation (Table 2) (Chun et al., 2018). These finding were in line with the phylogenomic studies revealing that the studied strains form five evolutionary lineages, which were distinct from those of the validly published species of *Streptomyces*. The strains can be differentiated from one another and from *Streptomyces* species validly named using the phenotypic, chemotaxonomic, phylogenomic, and genomic features.

**TABLE 2:** ANI and dDDH (*d*_4_ formula) values between the whole-genome sequence of the strains and their closest phylogenomic relatives.

Query strain (^T^)	Subject strain	dDDH (%)	ANI score (%)	G + C content difference (in %)
Strain DSM 40907	*Streptomyces nojiriensis* JCM 3382^T^	56.7	97.40	0.29
Strain DSM 40907	*Streptomyces spororaveus* NBRC 15456^T^	50.5	92.96	0.08
Strain DSM 40907	*Streptomyces avidinii* JCM 4726^T^	45.0	91.67	0.20
Strain DSM 40907	*Streptomyces avidinii* DSM 40526^T^	45.0	91.69	0.20
Strain DSM 40484	*Streptomyces dioscori* A217	45.0	91.57	0.09
Strain DSM 40484	*Streptomyces tauricus* JCM 4837^T^	43.7	91.23	0.02
Strain DSM 40484	*Streptomyces glomeroaurantiacus* JCM 4677^T^	35.2	87.72	0.51
Strain DSM 40976	*Streptomyces gardneri* JCM 4375^T^	44.4	91.33	0.32
Strain DSM 40976	*Streptomyces narbonensis* JCM 4147^T^	42.9	90.75	0.08
Strain DSM 40713	*Streptomyces cacaoi* subsp. *asoensis* JCM 4185^T^	37.8	88.96	1.01
Strain DSM 40713	*Streptomyces rishiriensis* NBRC 13407^T^	34.8	87.76	0.45
Strain DSM 40713	*Streptomyces humidus* JCM 4386^T^	34.1	87.48	0.43
Strain DSM 40971	*Streptomyces marinus* DSM 41968^T^	29.2	85.05	0.63
Strain DSM 40713	Strain DSM 40484^T^	24.7	80.89	0.52
Strain DSM 40976	Strain DSM 40907^T^	23.2	79.45	0.32
Strain DSM 40976	Strain DSM 40484^T^	22.8	78.59	1.18
Strain DSM 40976	Strain DSM 40713^T^	22.7	78.42	0.66
Strain DSM 40484	Strain DSM 40907^T^	22.6	77.93	0.86
Strain DSM 40713	Strain DSM 40907^T^	22.4	77.94	0.35
Strain DSM 40971	Strain DSM 40907^T^	21.4	75.97	0.36
Strain DSM 40713	Strain DSM 40971^T^	21.3	75.88	0.01
Strain DSM 40971	Strain DSM 40484^T^	21.3	75.89	0.50
Strain DSM 40976	Strain DSM 40971^T^	21.2	76.23	0.67

### 3.2 Antimicrobial potential of the five novel *Streptomyces* species

#### 3.2.1 *In silico* screening for secondary metabolites

The whole-genome-based phylogenetic classification of the five novel species and their closest neighbors revealed a clear grouping in five clades (Figure 2). The biosynthetic potential of the strains was unveiled by bioinformatics analysis of the genome sequences with antiSMASH. The strains within the five clades had a slightly different overall content of BGCs. The clade, which harbored DSM 40484^T^, showed the highest overall BGC content, with, on average, ∼37 BGCs, followed by the clades including DSM 40713^T^ (34 BGCs), DSM 40907^T^ (32 BGCs), DSM 40976^T^ (31 BGCs), and DSM 40971^T^ (27 BGCs) (Figure 2). Of all strains, *S. cacaoi* subsp*. asoensis* DSM 41440^T^ showed the highest BGC content, with 52 BGCs in total, while its closest phylogenetic relative, strain DSM 40713^T^, had 31 BGCs. The individual BGC contents of the four residual novel *Streptomyces* species were 33 BGCs for DSM 40907^T^, 32 BGCs for DSM 40976^T^, 36 BGCs for DSM 40484^T^, and 21 BGCs for DSM 40971^T^. A comparison of the number and classes of BGCs identified in the genomes did not reveal significant differences within and across the five clades due to the large BGC number variation intra-clade. However, there are some notable findings worth highlighting: the clade including DSM 40976^T^ showed low intra-clade variation in BGC number, while strain DSM 10713^T^ had only approximately half the BGCs than the closest neighbor *S. cacaoi* subsp*. asoensis* DSM 41440^T^. Finally, the clade to which DSM 40907^T^ belongs had an abundance of strains without any “ectoine” BGC, which may indicate a particular physiology or ecological niche of the respective microorganisms.

#### 3.2.2 *In vitro* screening for antimicrobial compounds

**FIGURE 3:**
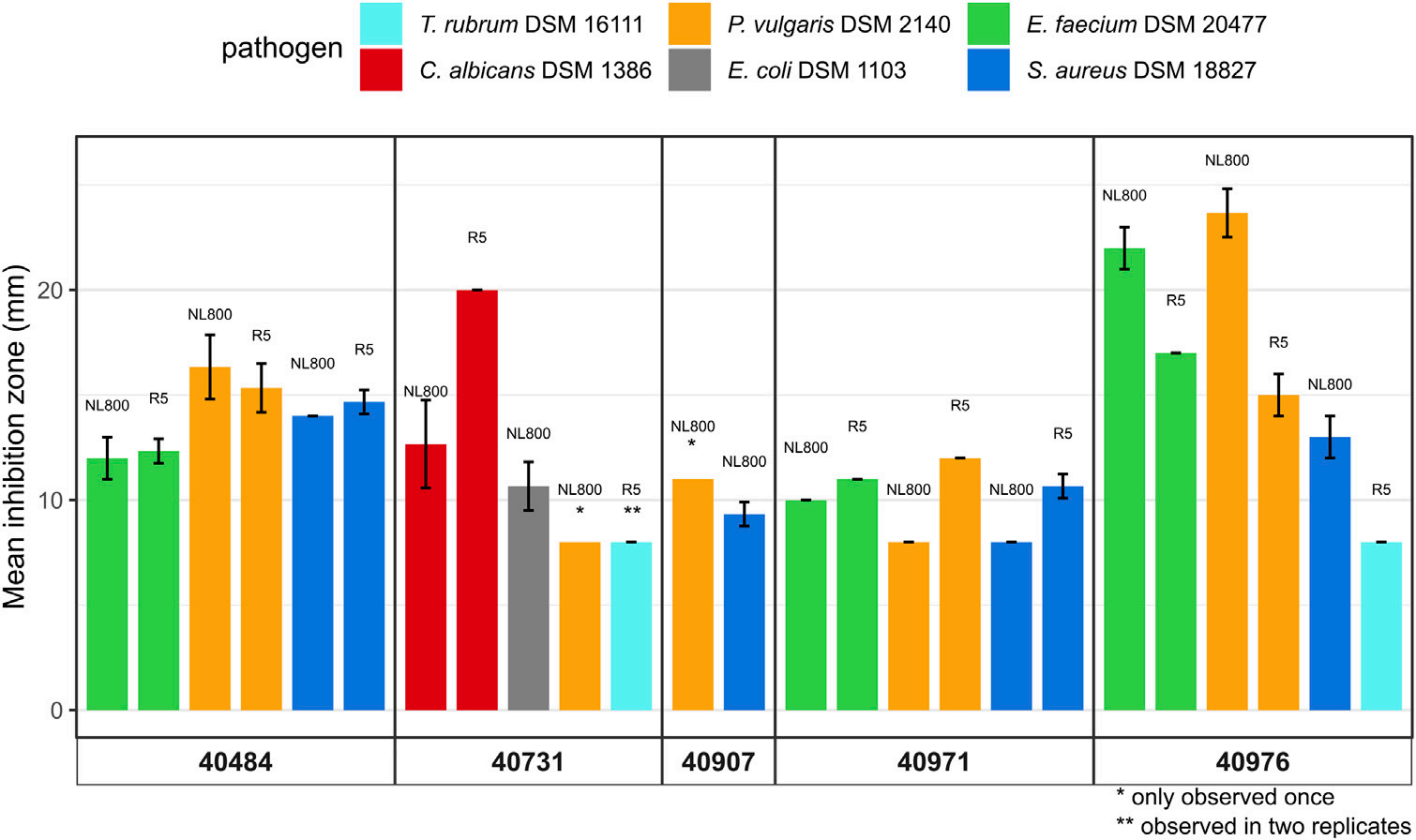
Antimicrobial bioassays with extract samples obtained from DSM 40484^T^, DSM 40713^T^, DSM 40907 ^T^, DSM 4097 1 ^T^, and DSM 40976^T^ grown for 72 h in R5 and NL800 medium against pathogenic test microorganisms, including *Staphylococcus aureus* DSM 18827 (dark blue), *Enterococcus faecium* DSM 20477 (green), *Escherichia coli* DSM 1103 (gray), *Proteus vulgaris* DSM 2140 (orange), *Candida albicans* DSM 1386 (red), and *Trichophyton rubrum* DSM 16111 (light blue). Inhibition zone diameters of bioassays are given in mm. Data shown are as the result of three independent biological replicates.

To assess the antibiotic production potential of the five novel *Streptomyces* species, extract samples were used to test for the antimicrobial activity in bioassays against a panel of selected strains from the WHO priority list, including Gram-positive and Gram-negative test bacteria, as well as against yeast and fungal test strain. Extracts obtained from cultures of DSM 40484^T^, DSM 40971^T^, and DSM 40976^T^ led to inhibition zones against the Gram-positive pathogens *S. aureus* DSM 18827 and *E. faecium* DSM 20477 and the Gram-negative strain *P. vulgaris* DSM 2140 (Figure 3). Extract samples from DSM 40713^T^ led to inhibition zones against *E. coli* DSM 1103 and *P. vulgaris* DSM 2140 and, thus, showed Gram-negative-specific antibiotic activity, whereas extract samples from DSM 40907^T^ led to inhibition against *S. aureus* DSM 18827 and *P. vulgaris* DSM 2140. Altogether, the culture extracts from the five novel species showed activity against at least two pathogenic test strains.

#### 3.2.3 Antibiotic production profile of novel *Streptomyces* species

Strain DSM 40713^T^ is an old deposit from a well-studied strain collection (Tübingen actinomycetes strain collection) known to be a ferrimycin producer. A ferrimycin gene cluster was identified (Supplementary Figure S3, region 16.1), where 100% of genes showed similarity to a known desferrioxamine B BGC from *Streptomyces griseus* subsp. *griseus* NBRC 13350 (MIBiG accession BGC0000941; Ohnishi et al., 2008) (Supplementary Figure S4). It was assumed that the strain was thoroughly investigated for antibiotic production and, therefore, DSM 40713^T^ was excluded from further substance analysis. DSM 40971^T^ was not further analyzed due to the low overall BGC content, as outlined in Supplementary Figure S5.

#### 3.2.4 Detection of cinerubin B in DSM 40484^T^

**FIGURE 4:**
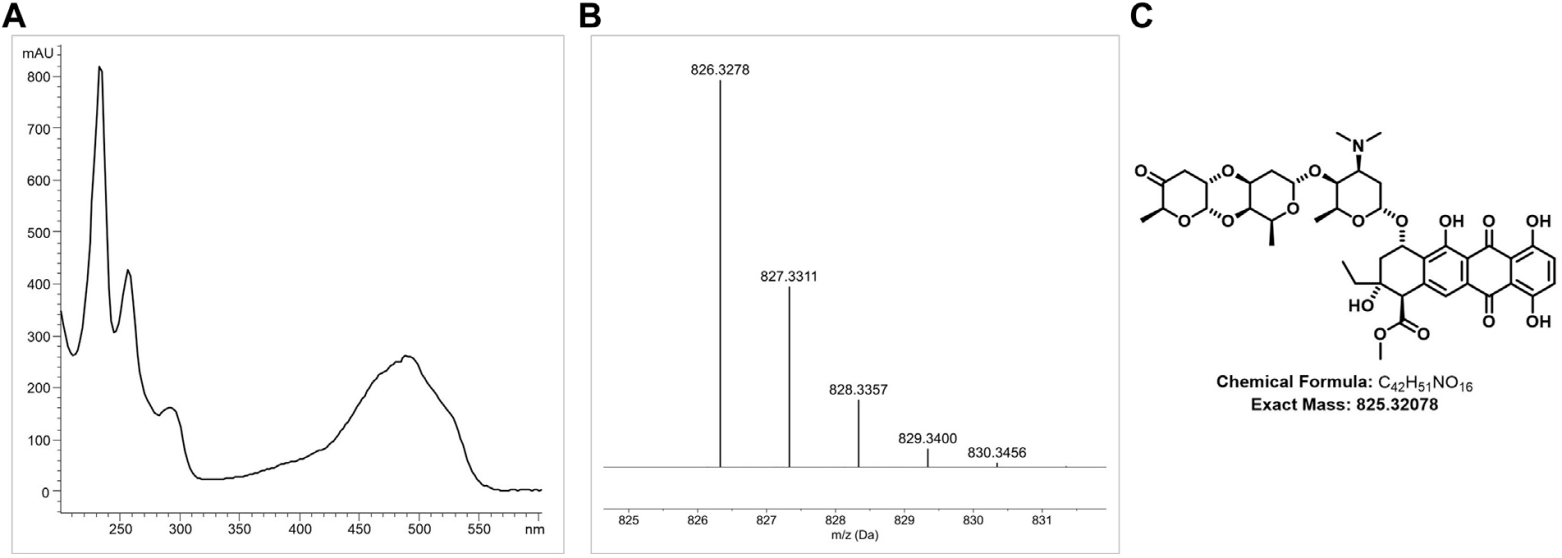
Detection of cinerubin B production in ethyl acetate extract samples of DSM 40484^T^ by LC-M analysis. Characteristic LC-HRMS analysis of cinerubin B **(A)**. HRMS data of the DSM 40484^T^ sample with a protonated molecular ion (*m/z*) of 825.3278 [M + H]^+^ **(B)**. Chemical structure of cinerubin B (C_42_H_51_NO_16_) **(C)**.

**FIGURE 5:**
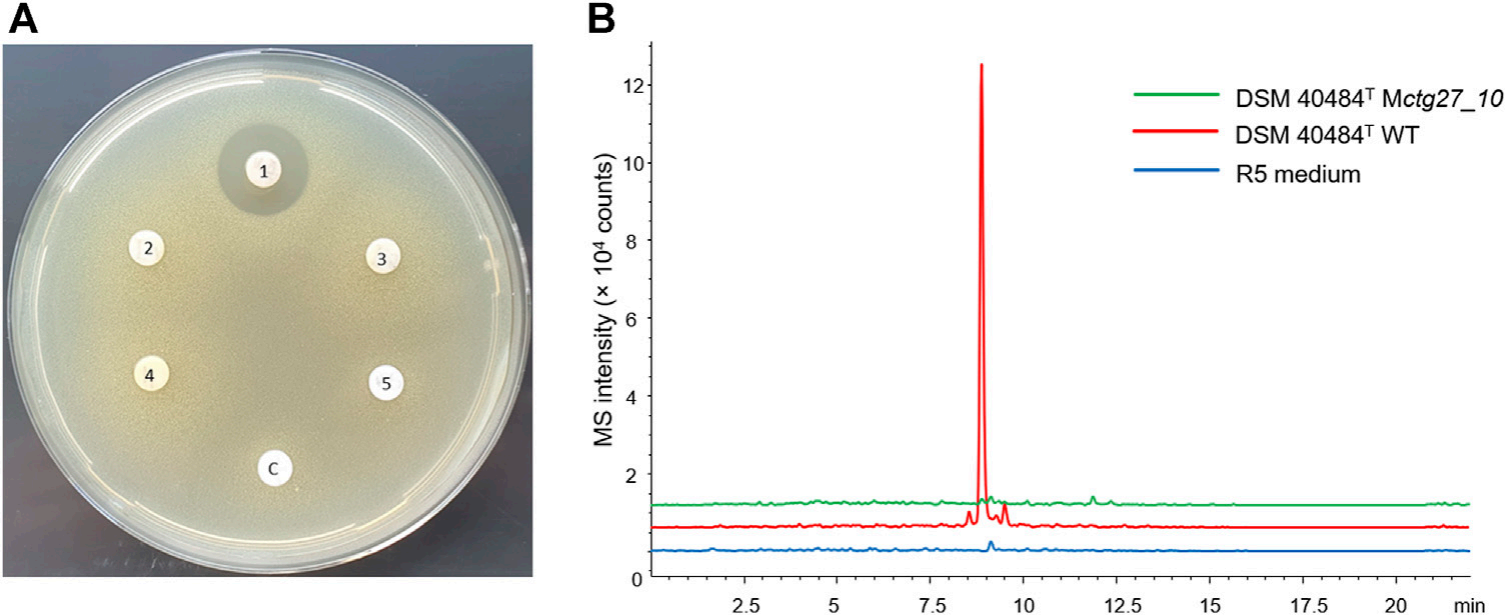
Inactivation of *ctg27_10* completely blocks cinerubin B production in DSM 40484^T^. **(A)** *K. rhizophila* DSM 11926^T^ growth inhibition around paper discs saturated with extracts from different cinerubin B producers: DSM 40484^T^ WT (1), *Mctg27_10* (2–4), extract from the fermentation medium R5 (5), negative control, and methanol (C). **(B)** Overlaid extracted ion chromatograms for the protonated molecular ion of cinerubin B (*m/z* [M+H]^+^ = 826.3) derived from DSM 40484^T^ strains grown in R5.

HPLC analysis of ethyl acetate extract samples from DSM 40484^T^ revealed a prominent peak at 240 nm with a retention time (RT) of 12.75, with a characteristic UV/Vis spectrum (Figure 4A). LC–HRMS analysis of a bioactive fraction sample of the DSM 40484^T^ extract delivered an exact mass of 825.3278 (Figure 4B), which matched with a sum formula of C_42_H_51_NO_16_ (Figure 4C). The characteristic UV/Vis spectrum (λ_max_ = 235, 255, 290, and 490 nm) matched with cinerubin B in an “in-house” UV/Vis database, which is a known glycosylated polyketide antibiotic (Ettlinger et al., 1959). Bioinformatics analysis of the genome sequence of DSM 40484^T^ led to the identification of cluster region 27.1, which showed similarity to a cinerubin BGC (Supplementary Figure S6). Sequence comparisons revealed that cluster region 27.1 contained all the genes suggested to be part of a functional cinerubin BGC, since 100% of the genes from the deposited cinerubin MIBiG reference sequence (BGC0000212) (Kersten et al., 2013) were also present in cluster region 27.1 (Supplementary Figure S7). To prove that the identified cluster region 27.1 from DSM 40484^T^ is responsible for cinerubin B biosynthesis, we inactivated the *ctg27_10* coding sequence, encoding a putative β-ketoacyl synthase, which was expected to be essential for aromatic polyketide biosynthesis (Supplementary Figure S7). Culture extracts from the *Streptomyces* sp. DSM 40484^T^ *Mctg27_10* mutant and the *Streptomyces* sp. DSM 40484^T^ wild-type (WT) strain were analyzed in comparative HPLC–MS and antibiotic activity tests against *K. rhizophila* DSM 11926^T^ as the test organism. In contrast to the WT strain, no antibiotic activity was detected from the extracts of the generated mutant strains (Figure 5A). Moreover, no mass peak corresponding to the molecular ion of cinerubin B (*m/z* [M+H]^+^ = 826.3) was present in the extracts of DSM 40484^T^ *Mctg27_10* (Figure 5B), indicating that the inactivation of *ctg27_10* completely abolished cinerubin B production in DSM 40484^T^. Until now, this is the first genetic evidence of the functionality of a cinerubin BGC by genetic knockout.

#### 3.2.5 Detection of a phosphonate antibiotic in DSM 40907^T^

**FIGURE 6:**
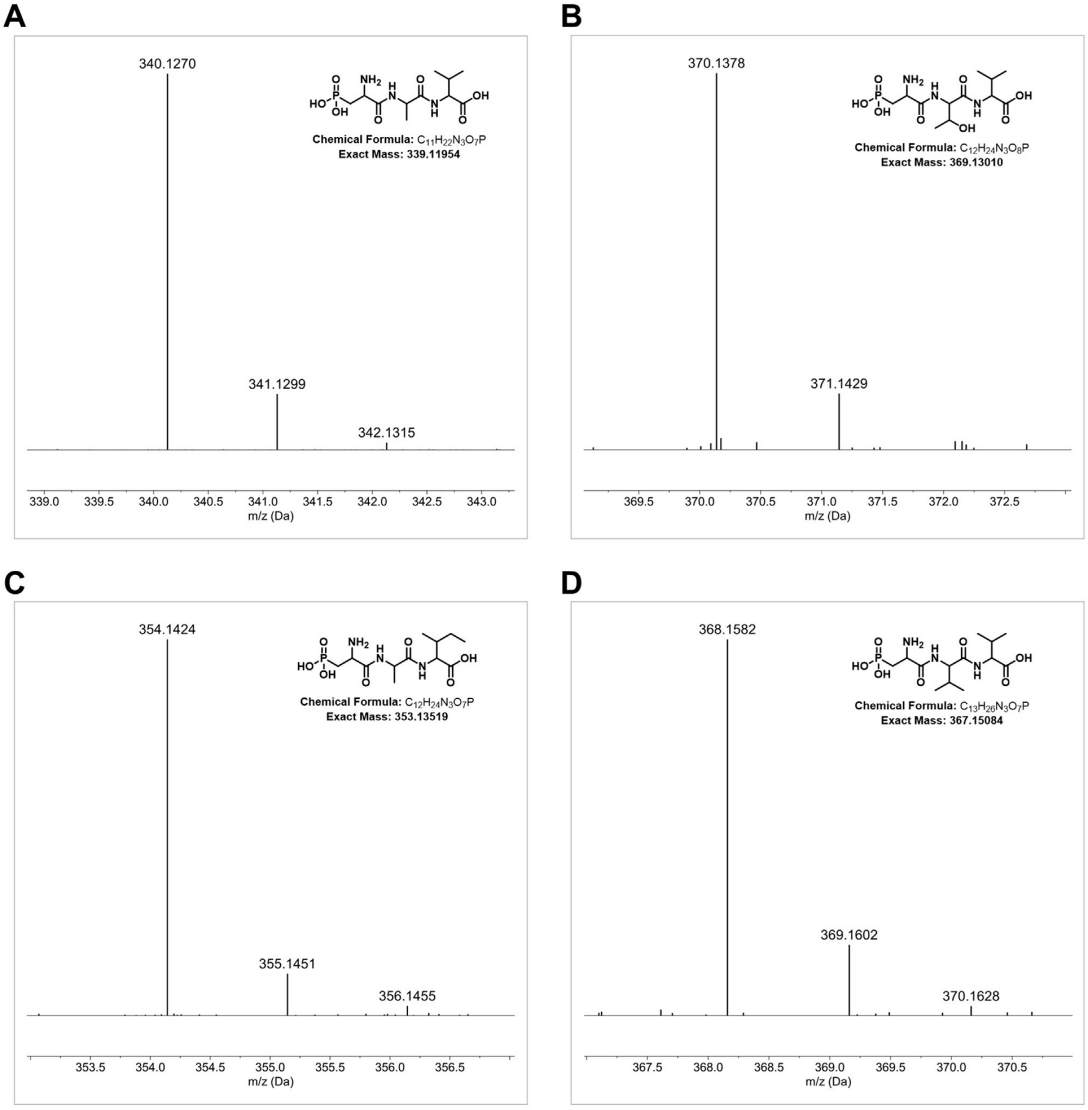
Detection of phosphonoalamides production in samples of DSM 40907T by HRMS analysis. HRMS data of the DSM 40907T sample with *m/z* 340.1268 [M + H]^+^ (C_11_H_23_N_3_O_7_P,) **(A)**, *m/z* 370.1378 [M + H]^+^ (C_12_H_25_N_3_O_8_P) **(B)**, *m/z* 354.1424 [M + H]^+^ (C_12_H_25_N_3_O_7_P) **(C)**, and *m/z* 368.1582 [M + H]^+^ (C_13_H_27_N_3_O_7_P) **(D)**.

Strain DSM 40907^T^ was identified as a potential phosphonate producer by BGC analysis (Supplementary Figure S8) due to the occurrence of a *pepM* coding sequence on region 3.1 (Supplementary Figure S9), which is indicative of phosphonate biosynthesis as it encodes a potential phosphoenolpyruvate mutase (PepM) known to be responsible for the first and essential biosynthetic step of phosphonate biosynthesis, converting phosphoenolpyruvate to phosphonopyruvate (Ju et al., 2015). Based on this assumption, culture supernatant samples from DSM 40907^T^ grown in GUBC medium (Parkinson et al., 2019) were analyzed with LC–HRMS. This led to the identification of characteristic mass fragmentations, including the protonated molecular ions *m/z* 340.1270 [M + H]^+^, 370.1378 [M + H]^+^, 354.1424 [M + H]^+^, and 368.1582 [M + H]^+^, which matched the sum formula of C_11_H_23_N_3_O_7_P, C_12_H_25_N_3_O_8_P, C_12_H_25_N_3_O_7_P, and C_13_H_27_N_3_O_7_P, respectively (Figures 6A–D, respectively). These data corresponded with the MS/MS fragmentation pattern reported for phosphonoalamides (Kayrouz et al., 2020), unveiling that DSM 40907^T^ produces phosphonoalamides as phosphonate antibiotics. Phosphonoalamides are phosphonoalanine-like natural compounds with good antibiotic activity against different Gram-positive and Gram-negative bacteria (Kayrouz et al., 2020), which is consistent with the observation that extract samples from DSM 40907^T^ showed bioactivity against *S. aureus* DSM 18827 and *P. vulgaris* DSM 2140 in bioassays, respectively (Figure 3).

#### 3.2.6 Identification of numerous SARP genes in DSM 40976^T^

**FIGURE 7:**
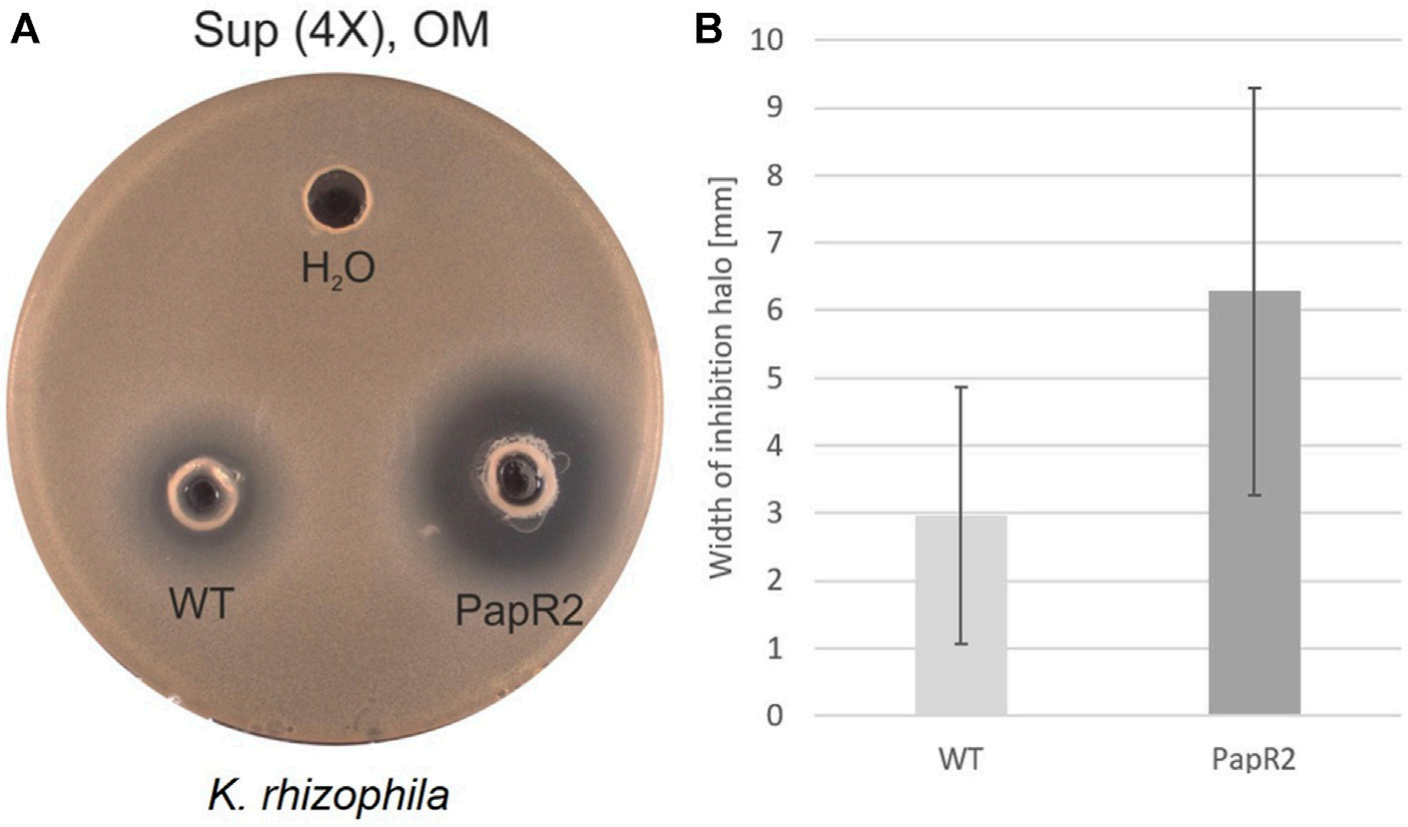
Bioassay results of *Streptomyces* sp. DSM 40976 WT and *papR2* overexpression strain (pGM1190/*papR2-tipA*p, “PapR2”) after 72 h cultivation in OM. **(A)** Supernatant (“Sup,” 4X-concentrated) was tested against *K. rhizophila* DSM 11926^T^, with H_2_O as the negative control. **(B)** Average width of inhibition halos against *K. rhizophila* DSM 11926^T^. Black lines show the standard deviation (number of replicates = 3).

The bioinformatics analysis of strain DSM 40976^T^ revealed another strain-specific feature, as it showed a comparatively large number of SARP (*Streptomyces* antibiotic regulatory protein) genes (eight in total) as part of BGCs (Supplementary Figure S10). Altogether, eight SARP genes were encoded in six of the 32 BGCs (Supplementary Figure S10). The six BGCs belonged to the following cluster types: type-I PKS, lanthipeptide-class, and three hybrid BGCs (Supplementary Figure S10). SARPs are transcriptional regulators, which act as pathway-specific activators of antibiotic biosynthesis (Bibb, 2005). In a previous study, we have shown that SARP regulators can be used as general activators of antibiotic biosynthesis in various actinomycetes when heterologously expressed (Krause et al., 2020). Due to the high abundance of SARP genes in a number of BGCs, DSM 40976^T^ was designated for heterologous SARP gene expression. For this purpose, the SARP-type regulator gene *papR2* from the pristinamycin producer *Streptomyces pristinaespiralis* was heterologously expressed in DSM 40976^T^ using the conjugatable, self-replicative plasmid pGM1190/*papR2*-*tipA*p. Both DSM 40976T pGM1190/*papR2*-*tipA*p and DSM 40976 WT strains were each grown in OM medium for 72 h. Ethyl acetate extracts and concentrated supernatant samples were obtained from DSM 40976^T^ cultures and were used for antimicrobial bioassays against *K. rhizophila* DSM 11926^T^. Here, specifically supernatant samples from DSM 40976 pGM1190/*papR2*-*tipA*p cultures resulted in a significantly improved bioactivity against *K. rhizophila* DSM 11926^T^ in comparison to samples from the DSM 40976^T^ WT (Figure 7). This indicates that the SARP expression in strain DSM 40976^T^ activated BGC expression and, thus, improved antibiotic production. The gene products of the five SARP genes from DSM 40976^T^ showed amino acid similarity scores of ≥ 50% compared to PapR2. The highest similarity score of 58% was found for a SARP amino acid sequence, which is encoded by the locus Tag: “ctg45_9”, located in region 45.1. Cluster region 45.1 represents a hybrid BGC, consisting of NRPS, NRPS-like, and type-I PKS core genes (Supplementary Table S6). Thus, gene region 45.1 is a potential BGC that may have been activated by PapR2 expression. However, due to the presence of several BGCs with *papR2* homologous genes in DSM 40976^T^, a reliable prediction of gene clusters that have been activated or bioactive compounds that were produced cannot be made. Definitely, the narrowing down to a few candidate gene clusters will help identify the bioactive compound in subsequent work.

#### 3.2.7 Description of *Streptomyces kutzneri* sp. nov.

*Streptomyces kutzneri* (kutz’ne.ri N.L. gen. n. *kutzneri*, referring to Professor Hans-Jürgen Kutzner, who contributed significantly to prokaryotic systematics) is a Gram-stain-positive, aerobic, non-motile bacterium that forms light-gray aerial mycelium on oatmeal agar medium that turns white on GYM medium and white-grayish on Bennett’s media. A dark-brown diffusible pigment is produced at an incubation temperature of 10°C and 15°C on GYM medium and at 28°C on ISP 7. The strain is able to grow from 10°C to 37°C, optimally at 28°C, and from pH 5–7.5, optimally at pH 7.0. Additional cultural and morphological properties are mentioned in Supplementary Table S1. It has *LL*-A2pm as the diamino acid of the cell wall peptidoglycan and diphosphatidylglycerol, phosphatidylethanolamine, phosphatidylinositol, a glycolipid, a lipid, two aminolipids, and four unidentified phospholipid components as the polar lipid profile. The fatty acid pattern (>4%) contains C_15:0_ *iso,* C_15:0_ *anteiso,* C_16:0_ *iso,* C_16:1_ CIS 9, C_16:0,_ C_17:0_ *iso,* and C_17:0_ *anteiso.* The menaquinone pattern (≥4%) encompasses MK-9 H_2_, MK-9 H_4_, MK-9 H_6_, and MK-9 H_8_. The genome size of the strain is 9.2 Mbp, and its *in silico* G + C content is 71.7%.

The type strain DSM 40907^T^ was isolated from soil of an unknown origin and deposited in the DSMZ culture collection. The genome sequences of DSM 40907T have been deposited in the DDBJ/ENA/GenBank databases under the accession number JASTTI000000000. The BioProject accession number is PRJNA980006.

#### 3.2.8 Description of *Streptomyces stackebrandtii* sp. nov.

*Streptomyces stackebrandtii* (stack.e.brandt’i.i. N.L. gen. n. *stackebrandtii*, referring to Prof. Erko Stackebrandt, a German microbiologist who has contributed significantly to the prokaryotic systematics) is a Gram-stain-positive, aerobic, non-motile bacterium that forms white–beige aerial mycelium on oatmeal agar and Bennett’s media that turns white on ISP 5 and ISP 7 media. The strain is able to grow from 10°C to 37°C, optimally at 28°C, and from pH 5–7.5, optimally at pH 7.0. Additional cultural and morphological properties are mentioned in Supplementary Table S1. It has *LL*-A2pm as the diamino acid of the cell wall peptidoglycan and diphosphatidylglycerol, phosphatidylethanolamine, phosphatidylinositol, a glycolipid, two aminolipids and lipids, and three unidentified phospholipid components as the polar lipid profile. The fatty acid pattern (>4%) contains C_15:0_ *iso,* C_15:0_ *anteiso,* C_16:0_ *iso,* C_16:1_ CIS 9, C_16:0,_ C_17:0_ *iso,* C_17:0_ *anteiso*, and C_17:0_ *cyclo* CIS 9. The menaquinone pattern (≥4%) encompasses MK-9 H_6_ and MK-9 H_8_. The genome size of the strain is 8.7 Mbp, and its *in silico* G + C content is 72.0%.

The type strain DSM 40976^T^ (=Gütt 467) was isolated from soil of an unknown origin and deposited in the DSMZ culture collection. The genome sequence of DSM 40976T has been deposited in the DDBJ/ENA/GenBank databases under the accession number JASTTG000000000. The BioProject accession number is PRJNA979996.

#### 3.2.9 Description of *Streptomyces zaehneri* sp. nov.

*Streptomyces zaehneri* (zaeh’ne.ri*.* N.L. gen. n. *zaehneri,* named after Professor Hans Zähner, a German microbiologist who contributed significantly to the field of natural products from microbial sources) is a Gram-stain-positive, aerobic, non-motile bacterium that forms gray-pinkish aerial mycelium on oatmeal agar that turns white–gray and gray on Bennett’s and ISP 7 media, respectively. A dark-brown diffusible pigment is produced on ISP 7 and GYM media at 28°C at 15°C, respectively. The strain is able to grow from 15°C to 37°C, optimally at 28°C, and from pH 5–7.5, optimally at pH 7.0. Additional cultural and morphological properties are mentioned in Supplementary Table S1. It has *LL*-A2pm as the diamino acid of the cell wall peptidoglycan and diphosphatidylglycerol, phosphatidylethanolamine, phosphatidylinositol, a glycolipid, two aminolipids and lipid, and four unidentified phospholipid components as the polar lipid profile. The fatty acid pattern (>4%) contains C_15:0_ *iso,* C_15:0_ *anteiso,* C_16:0_ *iso,* C_16:1_ CIS 9, C_16:0,_ C_17:0_ *iso,* and C_17:0_ *anteiso.* The menaquinone pattern (≥4%) encompasses MK-9 H_2_, MK-9 H_4_, MK-9 H_6_, and MK-9 H_8_. The genome size of the strain is 9.1 Mbp, and its *in silico* G + C content is 71.3%.

The type strain DSM 40713^T^ (= ETH 21510 = Tü 43) was isolated from soil from Firnhüttealp, Switzerland, and deposited by Prof. Hans Zähner in the DSMZ culture collection. The genome sequences of DSM 40713T have been deposited in the DDBJ/ENA/GenBank databases under the accession number JASTTJ000000000. The BioProject accession number is PRJNA980070.

#### 3.2.10 Description of *Streptomyces kroppenstedtii* sp. nov.

*Streptomyces kroppenstedtii* (krop.pen.stedt’i.i*.* N.L. gen. n. *kroppenstedtii,* named after Professor Reiner M. Kroppenstedt, a German microbiologist who is well known for his contribution to the bacterial taxonomy and contributed significantly to the Actinobacteria collection in the DSMZ culture collection) is a Gram-stain-positive, aerobic, non-motile bacterium that forms gray–orange aerial mycelium on oatmeal agar medium that turns gray–beige and white on ISP 7 and Bennett’s media, respectively. Bacteria diffusible pigment is produced at an incubation temperature of 10°C–28°C on GYM medium. The strain is able to grow from 10°C to 37°C, optimally at 28°C, and from pH 5–7.5, optimally at pH 7.0. Additional cultural and morphological properties are mentioned in Supplementary Table S1. It has *LL*-A2pm as the diamino acid of the cell wall peptidoglycan and diphosphatidylglycerol, phosphatidylethanolamine, phosphatidylinositol, an aminolipid, a glycolipid, a lipid, and four unidentified phospholipid components as the polar lipid profile. The fatty acid pattern (>4%) contains C_15:0_ *iso,* C_15:0_ *anteiso,* C_16:0_ *iso,* C_16:1_ CIS 9, C_16:0,_ C_17:0_ *iso,* and C_17:0_ *anteiso.* The menaquinone pattern (≥4%) encompasses MK-9 H_2_, MK-9 H_4_, MK-9 H_6_, and MK-9 H_8_. The genome size of the strain is 9.2 Mbp, and its *in silico* G + C content is 70.8%.

The type strain DSM 40484^T^ was isolated from soil of an unknown origin and deposited in the DSMZ culture collection. The genome sequences of DSM 40484T have been deposited in the DDBJ/ENA/GenBank databases under the accession number JASTTK000000000. The BioProject accession number is PRJNA980075.

#### 3.2.11 Description of *Streptomyces winkii* sp. nov.

*Streptomyces winkii* (win’ki.i. N.L. gen. n. *winkii*, named after Joachim Wink, a German microbiologist who has made significant contributions to actinobacterial systematics and natural products research) is a Gram-stain-positive, aerobic, non-motile bacterium that forms white-grayish aerial mycelium on oatmeal agar medium that turns white and black–brown on ISP 6, ISP 7, and Bennett’s media, respectively. A dark diffusible pigment is produced on ISP 6 and ISP 7 at 28°C. The strain is able to grow from 20°C to 37°C, optimally at 28°C, and from pH 5–7.5, optimally at pH 7.0. Additional cultural and morphological properties are mentioned in Supplementary Table S1. It has *LL*-A2pm as the diamino acid of the cell wall peptidoglycan and diphosphatidylglycerol, phosphatidylethanolamine, phosphatidylinositol, an aminolipid, a glycolipid, a lipid, and four unidentified phospholipid components as the polar lipid profile. The fatty acid pattern (>4%) contains C_15:0_ *iso,* C_15:0_ *anteiso,* C_16:0_ *iso,* C_16:1_ CIS 9, C_16:0,_ C_17:0_ *iso,* and C_17:0_ *anteiso.* The menaquinone pattern (≥4%) encompasses MK-9 H_2_, MK-9 H_4_, MK-9 H_6_, MK-9 H_8_, and MK-8 H_4_. The genome size of the strain is 7.2 Mbp, and its *in silico* G + C content is 71.3%.

The type strain DSM 40971^T^ (= Sandoz 59283) was isolated from soil of an unknown origin and deposited in the DSMZ culture collection. The genome sequences of DSM 40974T have been deposited in the DDBJ/ENA/GenBank databases under the accession number JASTTH000000000. The BioProject accession number is PRJNA980003.

## Data Availability

The datasets presented in this study can be found in online repositories. The names of the repository/repositories and accession number(s) can be found in the article/Supplementary Material.
