# Supplementary material for: Challenging old microbiological treasures for natural compound biosynthesis capacity
Source: Front Bioeng Biotechnol. 2024 Feb 1;12:1255151. doi: 10.3389/fbioe.2024.1255151 (PMC10867783; doi:10.3389/fbioe.2024.1255151)
Supplement: Supplementary file 1 [file DataSheet1.PDF]

Challenging old microbiological treasures for natural compound biosynthesis capacity

Imen Nouioui[1], Alina Zimmermann[1,2], Oliver Hennrich[1], Shuning Xia[2,3], Oona Rössler[1], Roman Makitrynskyy[1], Juan Pablo Gomez-Escribano[1], Gabriele Pötter[1], Marlen Jando[1], Meike Döppner[1], Jacqueline Wolf[1], Meina Neumann-Schaal[1,4], Chambers Hughes[2,3], Yvonne Mast[1,2,4,5]*

[1]Department Bioresources for Bioeconomy and Health Research, Leibniz Institute DSMZ - German Collection of Microorganisms and Cell Cultures, Inhoffenstraße 7B, 38124 Braunschweig, Germany

[2]German Center for Infection Research (DZIF), Partner Site Tübingen, Tübingen, Germany

[3]Department of Microbiology/Biotechnology, Interfaculty Institute of Microbiology and Infection Medicine, Faculty of Science, University of Tübingen, Auf der Morgenstelle 28, D-72076 Tübingen, Germany

[4]Braunschweig Integrated Centre of Systems Biology (BRICS), Rebenring 56, 38106 Braunschweig, Germany

[5]Technische Universität Braunschweig, Institut für Mikrobiologie, Rebenring 56, 38106 Braunschweig, Germany

*Correspondence:
Prof. Dr. Yvonne Mast
yvonne.mast@dsmz.de

Running title: Novel strains from old collections

Key words: actinomycetes, *Streptomyces*, antibiotic, novel species, polyphasic taxonomy, biosynthetic gene cluster

Table S1. Cultural and growth properties of the strains

	**40907T**	**40713T**	**40976T**	**40971T**	**40484T**
Project	5	5	5	5	5
**Temperature tests**					
10	+	-	+	-	+
15	+	+	+	-	+
20	++	++	++	++	++
25	++	++	+ +	++	++
28	+++	+++	+++	++	+++
37	+	+	+	+	+
42	-	-	-	-	-
45	-	-	-	-	-
**Media tests**					
ISP1	+	+	++	+	++
Description IPS1	light brown substrate mycelium	grey light brown aerial mycelim	white beige aerial mycelium	beige white substrate mycelium	beige aerial mycelium (pig)
ISP2 (-$CaCO_3$)	+	-	+	++	++
Description ISP2	no good growth, white aerial mycelium	-	beige substrate mycelium	white aerial mycelium	brown beige aerial mycelium
ISP3	+++	+++	+++	++	+++
Description ISP3	whitish grey aerial mycelium	pinkish grey aerial mycelium	white beige aerial mycelium	white greyish aerial mycelium	grey brownish aerial mycelium
ISP4	++	++	++	++	++
Description ISP4	white aerial mycelium	greyish white aerial mycelium	beige yellowish substrate mycelium	white beige aerial myelium	orange aerial mycelium
ISP5	++	++	+++	+	++
Description ISP5	green-beige substrate mycelium	beige aerial mycelium	white aerial mycelium	yellow substrate mycelium	orange brown substrate mycelium
ISP6	+	++	++	++	++
Description ISP6	light brown substrate mycelium	beige aerial mycelium with dark brown pigment	beige substrate aerial mycelium	dark brown, pigment diffusible	grey beige substrate myceliun, pig
ISP7	+++	+++	+++	++	+++
Description ISP7	black grey aerial mycelium with black diffusible pigment	dark grey aerial mycelium with diffusible pigment	white aerial mycelium	black aerial mycelium with diffusible pigment	grey beige aerial mycelium
TSA	++	+	+++	+	++

Description TSA	beige substrate mycelium	beige substrate mycelium	beige substrate mycelium	beige substrate mycelium	beige substrate mycelium
NA	+++	++	+++	++	++
Description NA	white aerial mycelium	greyish white substrate mycelium	beige substrate mycelium	white aerial mycelium	grey white aerial mycelium
Bennett media	+++	+++	+++	+++	+++
Description BM	greyish white aerial mycelium	greyish white substrate mycelium	white beige aerial mycelium	white aerial mycelium	white aerial mycelium
R5	+++	+++	+++	++	+++
Description R5	grey aerial mycelium	grey aerial mycelium	beige greyish aerial mycelium	grey aerial mycelium	orange brown substrate mycelium
GYM	+++	+++	+++	++	+++
Description GYM	white aerial mycelium	grey aerial mycelium	white aerial mycelium	beige substrate mycelium	yellow to brown beige substrate mycelium

+, weak growth;++moderate growth; +++ good growth

Table S2. Isoprenoid profile of the studied strains and their phylogenomic relatives. Nd, not detected.

**Strains**	**MK-9H$_2$**	**MK-9H$_4$**	**MK-9H$_6$**	**MK-9H$_8$**	**MK-9H$_{10}$**	**MK-8H$_4$**	**MK-8H$_6$**
Streptomyces DSM 40907^T	6.5	18.9	61.8	7.6	nd	2.9	2.2
Streptomyces DSM 41655^T	8.1	18.8	62.6	7.3	nd	1.3	1.7
Streptomyces DSM 40976^T	0.5	3.4	66.1	26.8	nd	nd	3.2
Streptomyces DSM 40064^T	1.2	14	57.7	25.1	0.5	nd	1.5
Streptomyces DSM 40016^T	0.7	3.7	65	26.2	0.4	traces	3.3
Streptomyces DSM 40713^T	6	17.2	64.4	7.9	nd	2.1	2.4
Streptomyces DSM 41440^T	9.9	23.4	51.7	11.5	0.2	1.6	1.5
Streptomyces DSM 40971^T	11	21.2	55.1	5.4	nd	5.3	3.4
Streptomyces DSM 41968^T	2	2.2	5.1	78.4	5.5	2.3	0.5

Streptomyces DSM 40484^T	8.7	23.3	59.6	6	nd	0.8	1.5

Table S3. Fatty acids profiles of the studied strains and their phylogenomic neighbours

Fatty acid profiles	DSM 40907^T	DSM 41655^T	DSM 40976^T	DSM 40064^T	DSM 40016^T	DSM 40713^T	DSM 41440^T	DSM 40484^T	DSM 40560^T	DSM 40971^T	DSM 41968^T
$C_{14:0}$ *iso*	-	-	-	-	-	-	-	-	-	-	5.6
$C_{15:0}$	-	-	-	4.3	-	-	6.1	-	-	-	-
$C_{15:0}$ *iso*	12.6	10.3	8.7	11.1	8.9	11.1	6.2	14.8	8.7	12.1	20.6
$C_{15:0}$ *anteiso*	19.2	22.8	29.0	23.2	24.2	19.1	22.7	17.3	25.7	19.4	28.9
$C_{16:0}$ *iso*	15.4	22.1	7.6	10.5	15.1	17.8	8.5	15.0	19.8	15.2	21.0
$C_{16:1}$ CIS 9	4.9	5.6	-	5.1	-	4.4	8.5	4.6	-	5.2	-
$C_{16:0}$	11.7	6.6	10.6	9.9	6.7	11.3	22.1	12.6	8.2	11.8	-
$C_{17:1}$ *iso* CIS 9	-	-	-	4.1	4.4	-	-	-	-	-	-

$C_{17:1}$ *anteiso* CIS 9	-	-	-	-	5.1	-	-	-	-	-	-
$C_{17:0}$ *iso*	9.2	-	3.6	6.2	4.4	8.32	-	10.6	4.7	8.6	4.8
$C_{17:0}$ *anteiso*	9.9	9.5	16.9	12.0	13.7	10.3	8.8	8.32	14.3	9.8	10.1
$C_{17:0}$ *cyclo* CIS 9	-	-	4.1	-	-	-	-	-	-	-	-

Table S4. the 16S rRNA gene sequence similarity between the studied strains and their closest phylogenetic neighbours

**Studied strains**	**Reference strains**	**16S rRNA gene similarity (%)**	**GenBank accession numbers of reference strains**
**Strain DSM 40484**^{T}	Strain DSM 40907^{T}	97.3	JASTTI000000000
	Strain DSM 40713^{T}	98.5	JASTTJ000000000
	Strain DSM 40976^{T}	97.6	JASTTG000000000

	Strain DSM 40971^T	95.0	JASTTH000000000
	Streptomyces glomeroaurantiacus NBRC 15418	99.3	AB249983
	Streptomyces aurantiacus NBRC 13017	99.3	AB184259
	Streptomyces ederensis NBRC 15410	99.0	AB184658.2
	Streptomyces umbrinus NBRC 13091	98.8	AB184305
	Streptomyces phaeochromogenes NBRC 3180	98.8	AB18473
	Streptomyces tauricus JCM 4837	98.7	AB045879
	Streptomyces humidus NBRC 12877	98.7	AB184213
	Streptomyces cacaoi subsp. *asoensis* NRRL B-16592	98.6	DQ026644
**Strain DSM 40713^T**	Strain DSM 40484^T	98.5	JASTTK000000000
	Strain DSM 40907^T	98.3	JASTTI000000000
	Strain DSM 40976^T	97.9	JASTTG000000000
	Strain DSM 40971^T	95.4	JASTTH000000000
	Streptomyces humidus NBRC 12877	99.7	AB184213
	Streptomyces cacaoi subsp. *asoensis* NRRL B-16592	99.5	DQ026644

	Streptomyces rishiriensis NBRC 13407|	99.4	AB184383
	Streptomyces tauricus JCM 4837	98.8	AB045879
	Streptomyces glomeroaurantiacus NBRC 15418	98.6	AB249983
	Streptomyces aurantiacus NBRC 13017	98.6	AB184259
**Strain DSM 40907^{T}**	Strain DSM 40484^{T}	97.3	JASTTK000000000
	Strain DSM 40713^{T}	98.3	JASTTJ000000000
	Strain DSM 40976^{T}	98.7	JASTTG000000000
	Strain DSM 40971^{T}	95.6	JASTTH000000000
	Streptomyces xanthophaeus NRRL B-5414	100	JOFT01000080
	Streptomyces nojiriensis LMG 20094	100	AJ781355
	Streptomyces sporoaveus LMG 20313	100	AJ781370
	Streptomyces lavendulae subsp. *lavendulae* NRRL B-2774	99.9	JOEW01000098
	Streptomyces virginiae NRRL ISP-5094	99.3	JOAK01000082
	Streptomyces cinnamonensis NBRC 15873	99.3	AB184707
	Streptomyces cirratus NRRL B-3250	99.8	AY999794

	Streptomyces vinaceus NBRC 13425	99.8	AB184394
	Streptomyces sporoverrucosus NBRC 15458	99.8	AB184684
	Streptomyces goshikiensis NBRC 12868	99.8	AB184204
	Streptomyces colombiensis NRRL B-1990	99.8	DQ026646
	Streptomyces subrutilus DSM 40445	99.7	X80825
	Streptomyces avidinii NBRC 13429	99.7	AB184395
	Streptomyces racemochromogenes NRRL B-5430	99.1	DQ026656
	Streptomyces polychromogenes NBRC 13072	99.1	AB184292
	Streptomyces flavotricini NRRL B-5419	99.1	JNXV01000042
	Streptomyces yangpuensis fd2-tb	99.1	LBMK01000002
	Streptomyces amritsarensis MTCC 11845	99.1	MQUR01000179
	Streptomyces lateritius LMG 19372	98.7	AJ781326
**Strain DSM 40971^T**	Strain DSM 40484^T	95.0	JASTTK000000000
	Strain DSM 40713^T	95.4	JASTTJ000000000
	Strain DSM 40976^T	95.3	JASTTG000000000

	Strain DSM 40907^T	95.6	JASTTI000000000
	Streptomyces nanshensis SCSIO 01066	99.3	EU589334
	Streptomyces abyssalis YIM M 10400	99.0	HQ585121
	Streptomyces xishensis YIM M 10378	98.5	HQ585118
**Strain DSM 40976**^{T}	Strain DSM 40484^T	97.6	JASTTK000000000
	Strain DSM 40713^T	97.9	JASTTJ000000000
	Strain DSM 40971^T	95.3	JASTTH000000000
	Strain DSM 40907^T	98.7	JASTTI000000000
	Streptomyces gardneri NBRC 12865	99.8	AB249908
	Streptomyces narbonensis NBRC 12801	99.8	AB184157
	Streptomyces zaomyceticus NBRC 13348	99.8	AB184346
	Streptomyces lateritius LMG 19372	99.7	NR_042293.1
	Streptomyces exfoliates NRRL B-2924	99.7	JNZP01000081
	Streptomyces venezuelae ATCC 10712	99.7	FR845719
	Streptomyces tanashiensis LMG 20274	99.7	AJ781362

	Streptomyces wedmorensis NRRL 3426	99.6	JNWK01000124
	Streptomyces litmocidini NBRC 12792	99.6	AB184149
	Streptomyces omiyaensis NBRC 13449	99.5	AB184411
	Streptomyces nashvillensis NBRC 13064	99.4	AB184286
	Streptomyces cinereoruber subsp. *cinereoruber* NBRC 12756	99.3	AB184121
	Streptomyces showdoensis NBRC 13417	99.3	AB184389
	Streptomyces viridobrunneus LMG 20317	99.3	AJ781372
	Streptomyces violaceorectus NBRC 13102	99.3	AB184314
	Streptomyces purpureus NBRC 13927	99.1	AB184547
	Streptomyces subrutilus DSM 40445	99.03	X80825

Table S5. Genomic features of the strains DSM 40907^T, DSM 40976^T, DSM 40713^T, DSM 40484^T, and DSM 40971^T.

	DSM 40907^T	DSM 40976^T	DSM 40713^T	DSM 40484^T	DSM 40971^T
Genome size (Mbp)	9.2	8.7	9.1	9.2	7.2
G+C content (%)	71.7	72.0	71.3	70.8	71.3
N50	402738	267261	255620	406403	838128
Number of coding sequences	8959	8204	8485	8356	6369
Number of RNAs	79	75	84	73	58

Table S6. BLASTP results from comparison of *Streptomyces* sp. DSM 40976^T SARPs with PapR2. SARP amino acids sequence with the highest homology to PapR2 is highlighted in grey.

Region	Locus tag	Identity	Similarity	Gaps
1.2	ctg1_159	40 %	47 %	5 %
3.1	ctg3_123	29 %	43 %	9 %
12.1	ctg12_93	37 %	50 %	3 %
12.1	ctg12_114	40 %	52 %	1 %
40.1	ctg40_15	39 %	51 %	5 %
44.1	ctg44_15	39 %	52 %	2 %
45.1	ctg45_9	43 %	58 %	2 %
45.1	ctg45_15	33 %	44 %	5 %

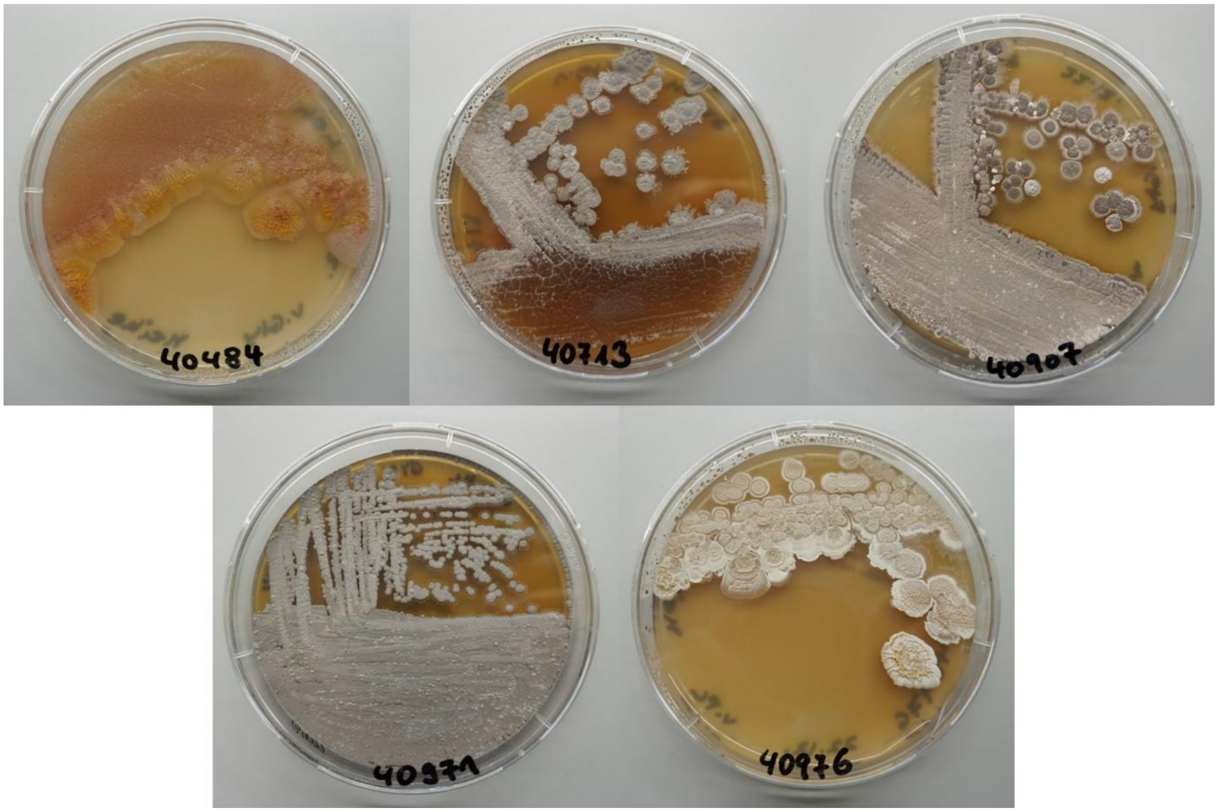

Figure S1: Pictures from strains DSM 40484^{T}, DSM 40713^{T}, DSM 40907^{T}, DSM 40971^{T}, and DSM 40976^{T} grown on R5 agar for 10 days at 28°C.

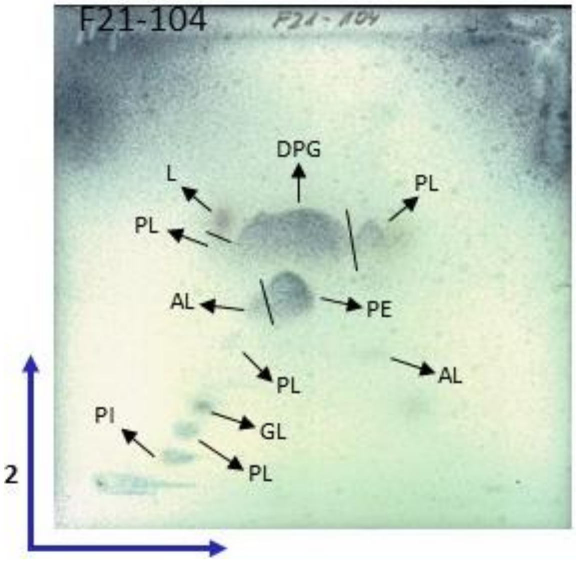
F21-104
DPG
L
PL
PL
AL
PE
PL
AL
PI
GL
PL
2

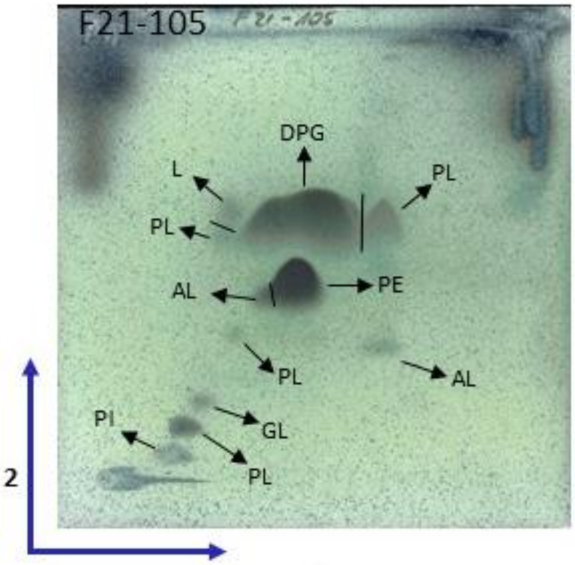
F21-105
DPG
L
PL
PL
AL
PE
PL
AL
PI
GL
PL
2

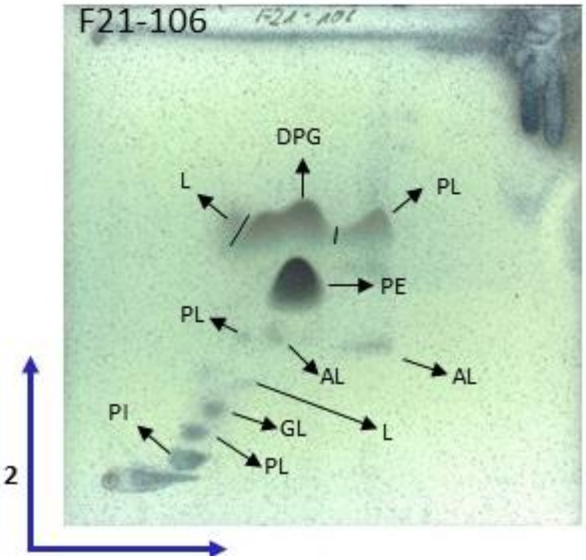
F21-106
DPG
L
PL
PE
PL
AL
AL
PI
GL
L
PL
2

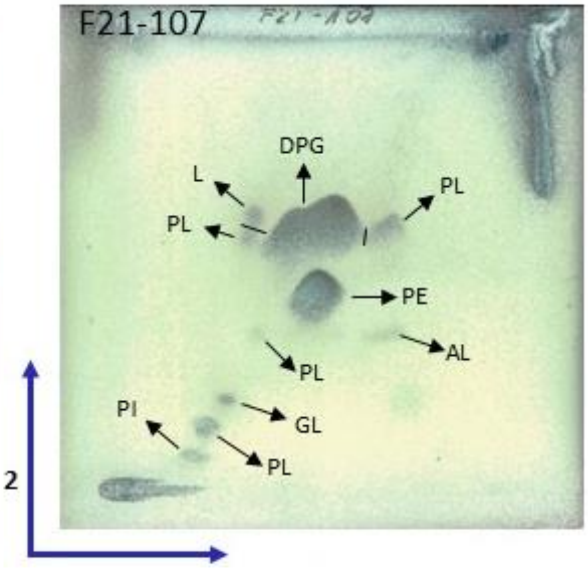
F21-107
DPG
L
PL
PL
PE
PL
AL
PI
GL
PL
2

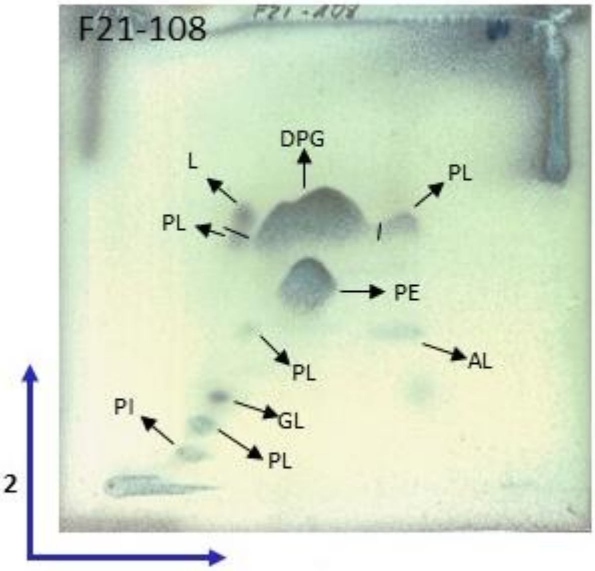
F21-108
DPG
L
PL
PL
PE
PL
AL
PI
GL
PL
2

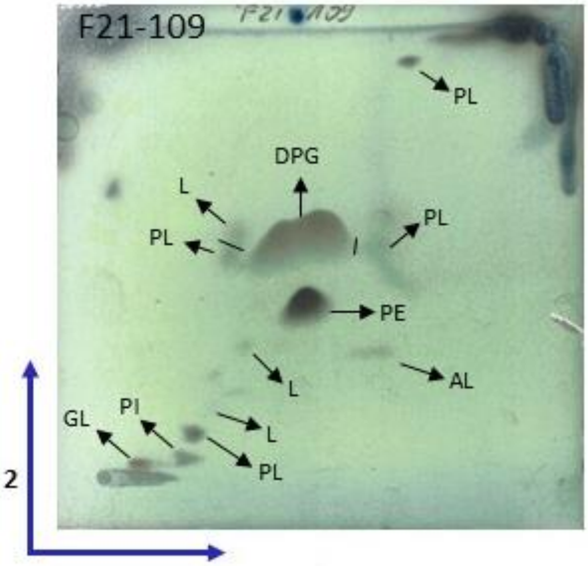
F21-109
PL
DPG
L
PL
PL
PE
L
AL
GL
PI
L
PL
2

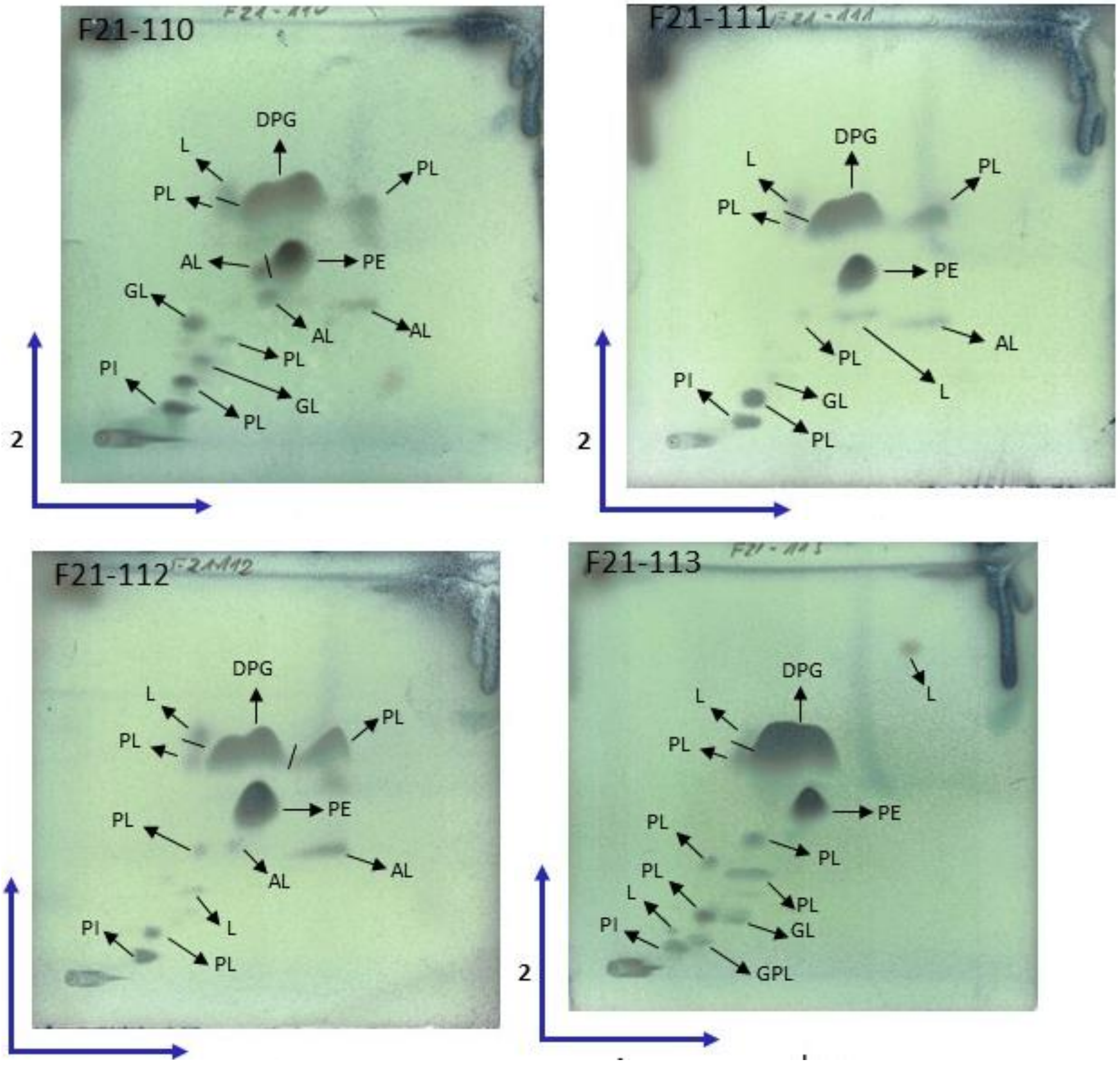

Figure S2. Two-dimensional TLC plates of polar lipids extracted from the strains DSM 40907^{T} (F21-104), DSM 41655^{T} (F21-109), DSM 40976^{T} (F21-106), DSM 40064^{T} (F21-110), DSM 40016^{T} (F21-111), DSM 40713^{T} (F21-105), DSM 41440^{T} (F21-112), DSM 40484^{T} (F21-108), DSM 40971^{T} (F21-107), DSM 41968^{T} (F21-113) stained with molybdatophosphoric acid (Sigma P1518). Key: AL, aminolipid, DPG, diphosphatidylglycerol; PE, phosphatidylethanolamine; PI, phosphatidylinositol; GPL, glycophospholipid; GL, glycolipid, L, lipid, PL, phospholipid. Solvent1: chloroform: methanol: distilled water (65:25:4 v/v/v/); solvent 2: chloroform: glacial acetic acid: methanol: distilled water (80:12:15:4 v/v/v).

Region	Type	From	To	Most similar known cluster		Similarity
Region 1.1	terpene	62,473	83,525	albaflavenone	Terpene	100%
Region 2.1	lassopeptide, RiPP-like	279,000	312,897	citrulassin D	RiPP	100%
Region 2.2	T2PKS	343,776	416,291	spore pigment	Polyketide	83%
Region 2.3	NRPS	468,079	518,346	thiazostatin / watasemycin A / watasemycin B / 2-hydroxyphenylthiazoline enantiopyochelin / isopyochelin	NRP	73%
Region 3.1	terpene	48,655	75,363	hopene	Terpene	92%
Region 3.2	T1PKS, hglE-KS	140,268	191,840	glycinocin A	NRP	11%
Region 3.3	RiPP-like, lanthipeptide-class-iii	440,743	468,402	informatipeptin	RiPP:Lanthipeptide	100%
Region 4.1	lanthipeptide-class-i	70,956	96,137			
Region 4.2	NRPS, siderophore	294,438	356,514	scabichelin	NRP	100%
Region 5.1	melanin	11,930	22,298	melanin	Other	57%
Region 5.2	amglyccycl	263,901	285,154	cetoniacytone A	Other:Cyclitol	12%
Region 5.3	NRPS-like, T1PKS	360,265	396,460	mycotrienin I	NRP + Polyketide	57%
Region 6.1	indole	91,848	113,125	5-isoprenylindole-3-carboxylate β-D-glycosyl ester	Other	23%
Region 6.2	nucleoside	194,847	215,548	Sch-47554 / Sch-47555	Polyketide	10%
Region 6.3	redox-cofactor	312,350	335,040			
Region 6.4	betalactone	361,037	381,691			
Region 8.1	NRPS	245,232	289,392	streptonigrin	Other	5%
Region 12.1	ectoine	152,577	162,981	ectoine	Other	100%
Region 14.1	NRPS-like, T1PKS, NRPS	2	101,439	antimycin	NRP + Polyketide	100%
Region 14.2	T1PKS	190,925	233,685	geldanamycin	Polyketide	17%
Region 16.1	siderophore	43,460	55,229	desferrioxamin B / desferrioxamine E	Other	83%
Region 16.2	melanin	159,441	169,923	istamycin	Saccharide	5%
Region 23.1	NAPAA	29,234	63,277			
Region 24.1	terpene	46,539	68,752	geosmin	Terpene	100%
Region 25.1	siderophore	80,923	92,863			
Region 26.1	RiPP-like	1	6,853			
Region 32.1	other, terpene, T3PKS	12,238	87,648	merochlorin A / merochlorin B / deschloro-merochlorin A / deschloro-merochlorin B / isochloro-merochlorin B / dichloro-merochlorin B / merochlorin D / merochlorin C	Terpene + Polyketide:Type III	56%
Region 38.1	T3PKS	1	23,693	germicidin	Other	100%
Region 40.1	T1PKS	1	42,276	rifamycin	Polyketide	30%
Region 53.1	T1PKS	1	14,725	angolamycin	Polyketide	80%
Region 55.1	butyrolactone	3,580	12,136	lactonamycin	Polyketide	7%

Figure S3: AntiSMASH output for strain DSM 40713^T. Desferrioxamine BGC is highlighted by a yellow arrow.

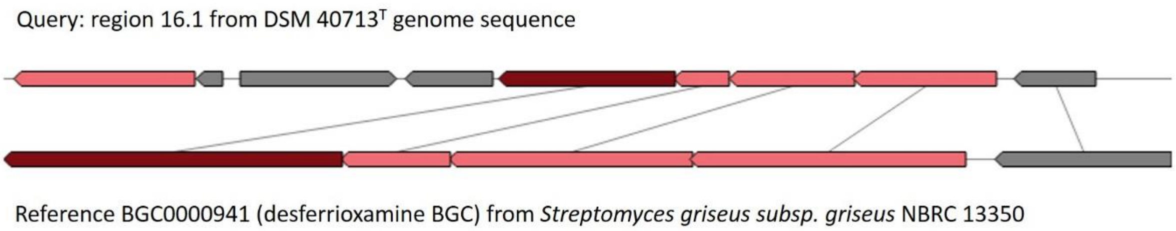

Figure S4: BGC comparison between region 16.1 from DSM 40713^T genome sequence and reference sequence BGC0000941 (desferrioxamine BGC) from *Streptomyces griseus subsp. griseus* NBRC 13350. Homologous genes are connected by grey lines. Colour code according to antiSMASH 6.0 (Blin et al., 2021).

Region	Type	From	To	Most similar known cluster		Similarity
Region 1.1	transAT-PKS	870,740	959,328	lactimidomycin / 8,9-dihydrolactimidomycin / 8-hydroxy-8,9-dihydrolactidomycin / 17-hydroxy-8-desmethoxy-isomigrastatin	Polyketide:Modular type I + Polyketide:Trans-AT type I	100%
Region 2.1	siderophore	267,919	281,542			
Region 2.2	linaridin	417,988	438,608	legonaridin	RiPP	16%
Region 2.3	NRPS-like	463,511	507,113	porothramycin A	NRP	5%
Region 2.4	ectoine	859,188	869,589	ectoine	Other	100%
Region 3.1	siderophore	1	8,624	desferrioxamine E	Other	100%
Region 3.2	butyrolactone	556,305	567,351	neocarzinostatin	Polyketide:Iterative type I + Polyketide:Enediyne type I	4%
Region 4.1	NRPS-like , T1PKS	1	35,866	candicidin	Polyketide	38%
Region 4.2	T3PKS	189,887	230,882	alkylresorcinol	Polyketide	66%
Region 4.3	terpene	468,878	495,519	hopene	Terpene	61%
Region 5.1	terpene	205,193	226,974	brasilicardin A	Terpene + Saccharide	30%
Region 5.2	RiPP-like	421,177	431,380			
Region 6.1	T1PKS , NRPS-like	153,065	199,203	amipurimycin	Polyketide	83%
Region 7.1	linaridin	116,898	137,617	pentostatine / vidarabine	Other	9%
Region 8.1	siderophore	60,590	75,730	ficellomycin	NRP	3%
Region 8.2	NRPS-like , NRPS	129,688	198,427	auroramycin	Polyketide	14%
Region 8.3	hglE-KS , T1PKS	330,548	382,477	FD-594	Polyketide	6%
Region 8.4	RiPP-like	470,185	481,555			
Region 10.1	T1PKS	1	119,250	nystatin-like Pseudonocardia polyene	Polyketide	47%
Region 11.1	terpene , T2PKS	109,809	204,014	WS79089A / hexaricin B / hexaricin C	Polyketide	33%
Region 14.1	T1PKS	1	17,937	aculeximycin	Polyketide	17%

Figure S5: AntiSMASH output for strain DSM 40971^{T}.

Region	Type	From	To	Most similar known cluster		Similarity
Region 1.1	terpene	53,905	78,421	hopene	Terpene	92%
Region 1.2	NRPS-like , NRPS , T1PKS , other , terpene	85,626	270,964	aurantimycin A	NRP + Polyketide	48%
Region 1.3	terpene	452,982	471,652	herboxidiene	Polyketide	4%
Region 1.4	T1PKS , terpene	500,786	544,949	oxalomycin B	NRP + Polyketide	9%
Region 1.5	RiPP-like	571,156	581,371	informatipeptin	RiPP:Lanthipeptide	42%
Region 2.1	NRPS	2	40,706	feglymycin	NRP	15%
Region 2.2	T2PKS , ladderane	91,032	167,482	simocyclinone D8	Saccharide + Polyketide:Modular type I + Polyketide:Type II + Other:Aminocoumarin	40%
Region 2.3	terpene	265,985	291,541	isorenieratene	Terpene	100%
Region 2.4	NRPS	303,312	380,731	borrelidin	Polyketide:Modular type I	6%
Region 2.5	NRPS	385,987	434,769	rimosamide	NRP	21%
Region 2.6	NRPS	590,601	634,950	diisonitrile antibiotic SF2768	NRP	55%
Region 4.1	siderophore	29,249	44,168			
Region 4.2	lanthipeptide-class-iv	167,477	190,230			
Region 4.3	NRPS , betalactone , NRPS-like	240,238	336,061	vazabitide A	NRP	21%
Region 4.4	RiPP-like	384,253	395,575			
Region 4.5	terpene	419,282	441,468	geosmin	Terpene	100%
Region 4.6	NAPAA	482,275	517,242	stenothricin	NRP:Cyclic depsipeptide	13%
Region 5.1	terpene	35,294	56,307	albaflavenone	Terpene	100%
Region 7.1	NRPS	11,957	55,991	lysolipin I	Polyketide	4%
Region 7.2	nucleoside	205,622	226,326			
Region 8.1	siderophore	309,734	321,521	desferrioxamin B / desferrioxamine E	Other	83%
Region 9.1	siderophore	266,407	279,796	grincamycin	Polyketide:Type II + Saccharide:Hybrid/tailoring	8%
Region 12.1	NRPS	170,047	226,930	foxicins A-D	NRP + Polyketide	29%
Region 13.1	T3PKS	135,551	176,669	alkylresorcinol	Polyketide	66%
Region 13.2	T1PKS	232,646	259,584	arsono-polyketide	Polyketide	37%
Region 16.1	T3PKS , NRPS	7,508	112,523	herboxidiene	Polyketide	10%
Region 20.1	RiPP-like	62,191	74,065			
Region 20.2	siderophore	147,794	161,085			
Region 21.1	NRPS , T3PKS , terpene	1	89,696	feglymycin	NRP	63%
Region 22.1	PKS-like , NRPS-like , T1PKS , ectoine	94,328	145,277	showdomycin	Other	17%
Region 26.1	ectoine	81,137	91,541	ectoine	Other	100%
Region 27.1	T2PKS , PKS-like , RRE-containing	1	45,070	cinerubin B	Polyketide:Type II	82%
Region 27.2	melanin	91,889	102,404	melanin	Other	100%
Region 29.1	PKS-like	1	21,238	arsono-polyketide	Polyketide	75%
Region 32.1	terpene	1	18,442	2-methylisoborneol	Terpene	100%
Region 33.1	NAPAA	1	24,019	rapamycin	NRP + Polyketide	14%

Figure S6: AntiSMASH output for strain DSM 40484^{T}. Cinerubin BGC is highlighted by a yellow arrow.

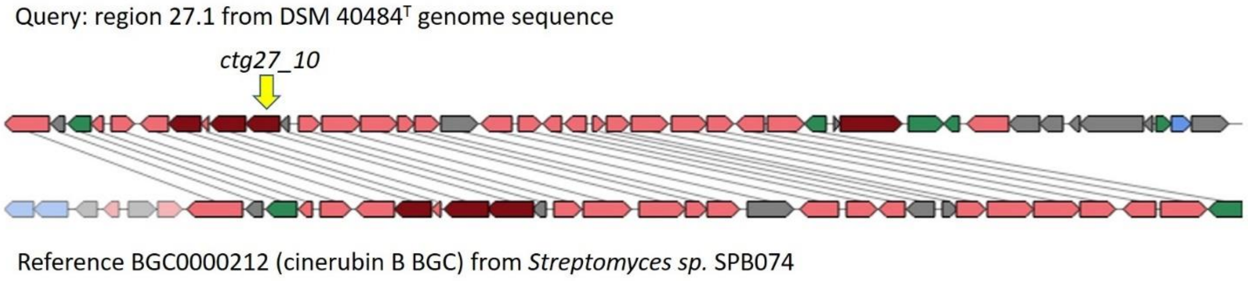

Figure S7: BGC comparison between region 27.1 from DSM 40484^{T} genome sequence and reference BGC0000212 (cinerubin B BGC) from *Streptomyces sp.* SPB074. Homologous genes are connected by grey lines. Colour code according to antiSMASH 6.0 (Blin et al., 2021). *ctg27_10*, the target gene for inactivation, is indicated by a yellow arrow.

Region	Type	From	To	Most similar known cluster		Similarity
Region 1.1	T2PKS	1	37,729	spore pigment	Polyketide	50%
Region 1.2	NRPS , T1PKS	578,207	643,359	coelichelin	NRP	72%
Region 1.3	butyrolactone	717,322	728,293	sanglifehrin A	NRP + Polyketide	4%
Region 2.1	NRPS-like , betalactone , NRPS	249,990	304,129	pepticinnamin E	NRP + Polyketide	10%
Region 2.2	RiPP-like	587,932	598,219			
Region 3.1	phosphonate	508	41,386	thioplatensimycin / thioplatencin	Terpene	4%
Region 3.2	CDPS	431,006	451,764	BD-12	NRP	17%
Region 4.1	terpene	327,079	347,524	ebelactone	Polyketide	5%
Region 4.2	terpene , lanthipeptide-class-iii	394,241	429,414	SapB	RiPP:Lanthipeptide	100%
Region 4.3	NRPS-like	452,684	496,001	lipstatin	NRP	42%
Region 4.4	lanthipeptide-class-iv	521,968	544,784	ikarugamycin	NRP + Polyketide:Iterative type I	12%
Region 5.1	nucleoside	57,527	77,895	toyocamycin	Other	40%
Region 6.1	terpene	154,601	181,664	hopene	Terpene	61%
Region 6.2	T1PKS , hglE-KS	249,404	300,804	lasalocid	Polyketide	9%
Region 6.3	NRPS	349,608	436,014	kirromycin	NRP + Polyketide:Modular type I + Polyketide:Trans-AT type I	22%
Region 7.1	NAPAA	50,678	84,589			
Region 7.2	CDPS	86,182	106,922			
Region 7.3	T3PKS	154,235	195,296	alkylresorcinol	Polyketide	100%
Region 7.4	siderophore	229,427	242,618			
Region 7.5	melanin	364,051	391,481	istamycin	Saccharide	4%
Region 7.6	terpene	393,948	414,853	monensin	Polyketide	5%
Region 9.1	RiPP-like	41,360	52,460	ashimides	NRP	12%
Region 9.2	RiPP-like	65,158	76,525			
Region 9.3	siderophore	307,754	322,776			
Region 12.1	terpene	17,853	38,944	cyclothiazomycin	RiPP:Thiopeptide	14%
Region 12.2	lanthipeptide-class-i	147,541	172,111			
Region 13.1	terpene	36,641	58,857	toxoflavin / fervenulin	Other	14%
Region 13.2	phenazine	156,570	177,079	lomofungin	Other	39%
Region 21.1	T1PKS , NRPS-like	6,488	71,082	prejadomycin / rabelomycin / gaudimycin C / gaudimycin D / UWM6 / gaudimycin A	Polyketide:Type II + Saccharide:Hybrid/tailoring	4%
Region 24.1	siderophore	58,966	70,747	desferrioxamin B	Other	100%
Region 28.1	butyrolactone	30,730	41,185			
Region 31.1	NRPS-like , NRPS	45,847	70,120	deimino-antipain	NRP	44%
Region 37.1	T1PKS	1	59,349	macrotermycins	Polyketide	61%

Figure S8: AntiSMASH output for strain DSM 40907^{T}. Phosphonate BGC is highlighted by a yellow arrow.

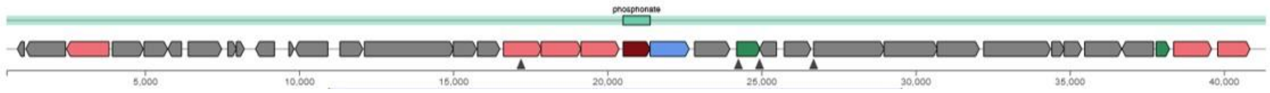

Figure S9: Genetic organization of potential phosphonate BGC encoded on region 3.1 in strain DSM 40907^T. The *pepM* gene is indicated as red arrow.

Region	Type	From	To	Most similar known cluster		Similarity
Region 1.1	melanin	122,835	133,248	istamycin	Saccharide	8%
Region 1.2	T1PKS	162,151	235,976	rosamicin / salinipyrone A / pacificanone A	Polyketide	60%
Region 1.3	NRPS-like	817,606	861,304	lankamycin	Polyketide	16%
Region 2.1	terpene	231,428	258,013	hopene	Terpene	69%
Region 2.2	T3PKS	524,459	565,616	alkylresorcinol	Polyketide	100%
Region 3.1	lanthipeptide-class-ii, CDPS	99,562	148,659	SBI-06990 A1 / SBI-06989 A2	RiPP:Lanthipeptide	100%
Region 3.2	NRPS, RiPP-like	191,907	256,783	salinichelins	NRP	53%
Region 5.1	RiPP-like	147,012	158,472			
Region 5.2	butyrolactone	273,644	284,630	macrotetrolide	Polyketide	33%
Region 7.1	NRPS, lanthipeptide-class-ii	1	30,268	friulimicin A / friulimicin B / friulimicin C / friulimicin D	NRP	15%
Region 7.2	lanthipeptide-class-ii	181,067	203,916			
Region 9.1	NAPAA	1	25,073	nocathiacin	RiPP:Thiopeptide	4%
Region 10.1	terpene	2	10,459			
Region 10.2	terpene	198,148	219,446	2-methylisoborneol	Terpene	100%
Region 11.1	NRPS	99,540	155,801	diazaquinomycin H / diazaquinomycin J	Other	4%
Region 12.1	T2PKS, PKS-like, RRE-containing, T1PKS	47,227	153,348	neocarzilin A / neocarzilin B	Polyketide	50%
Region 13.1	T3PKS	61,405	102,526	flaviolin	Other	75%
Region 13.2	terpene	119,850	142,045	geosmin	Terpene	100%
Region 13.3	ectoine	188,665	199,084	ectoine	Other	100%
Region 14.1	siderophore	34,612	46,402	desferrioxamin B	Other	100%
Region 17.1	butyrolactone	94,156	105,151			
Region 17.2	RiPP-like	146,548	158,845	divergolide A / divergolide B / divergolide C / divergolide D	Polyketide:Modular type I	6%
Region 22.1	T1PKS	1	31,140	ML-449	Polyketide:Modular type I	33%
Region 23.1	indole, NRPS-like	42,619	104,660	spiroindimicin A / spiroindimicin B / spiroindimicin C / spiroindimicin D / indimicin A / indimicin B / indimicin C / indimicin D / indimicin E / lynamicin A / lynamicin D / lynamicin F / lynamicin G	Other	21%
Region 31.1	siderophore	8,555	23,052	ficellomycin	NRP	3%
Region 40.1	other, NRPS-like, T1PKS, terpene	1	67,150	rubradirin	Polyketide	70%
Region 42.1	lanthipeptide-class-iv, other	898	54,130	venezuelin	RiPP:Lanthipeptide	100%
Region 44.1	NRPS-like, aminocoumarin, T1PKS	5,394	46,611	rubradirin	Polyketide	36%
Region 45.1	T1PKS, NRPS, NRPS-like	1	45,348	microtermolide A	NRP + Polyketide	40%
Region 62.1	T2PKS	1	6,934	spore pigment	Polyketide	41%
Region 63.1	NRPS	1	5,404			
Region 64.1	T1PKS	1	4,905	maklamicin	Polyketide	13%

Figure S10: AntiSMASH output for strain DSM 40976^T. Occurrence of SARP genes within a BGC is with yellow arrows.
